# Invited Review: APOE at the interface of inflammation, neurodegeneration and pathological protein spread in Alzheimer's disease

**DOI:** 10.1111/nan.12529

**Published:** 2018-11-28

**Authors:** M. Tzioras, C. Davies, A. Newman, R. Jackson, T. Spires‐Jones

**Affiliations:** ^1^ UK Dementia Research Institute and Centre for Discovery Brain Sciences The University of Edinburgh Edinburgh UK; ^2^ Massachusetts General Hospital and Harvard Medical School Charlestown MA USA

**Keywords:** Alzheimer's disease, APOE, Apolipoprotein E, glia, inflammation, neurodegeneration, tau

## Abstract

Despite more than a century of research, the aetiology of sporadic Alzheimer's disease (AD) remains unclear and finding disease modifying treatments for AD presents one of the biggest medical challenges of our time. AD pathology is characterized by deposits of aggregated amyloid beta (Aβ) in amyloid plaques and aggregated tau in neurofibrillary tangles. These aggregates begin in distinct brain regions and spread throughout the brain in stereotypical patterns. Neurodegeneration, comprising loss of synapses and neurons, occurs in brain regions with high tangle pathology, and an inflammatory response of glial cells appears in brain regions with pathological aggregates. Inheriting an apolipoprotein E ε4 (*APOE4*) allele strongly increases the risk of developing AD for reasons that are not yet entirely clear. Substantial amounts of evidence support a role for APOE in modulating the aggregation and clearance of Aβ, and data have been accumulating recently implicating APOE4 in exacerbating neurodegeneration, tau pathology and inflammation. We hypothesize that APOE4 influences all the pathological hallmarks of AD and may sit at the interface between neurodegeneration, inflammation and the spread of pathologies through the brain. Here, we conducted a systematic search of the literature and review evidence supporting a role for APOE4 in neurodegeneration and inflammation. While there is no direct evidence yet for APOE4 influencing the spread of pathology, we postulate that this may be found in future based on the literature reviewed here. In conclusion, this review highlights the importance of understanding the role of APOE in multiple important pathological mechanisms in AD.

## Introduction

The greatest genetic risk factor for sporadic AD is a polymorphism in the apolipoprotein E (*APOE)* gene. The *APOE* ε4 (*APOE4*) allele is associated with increasing risk of AD in a dose‐dependent manner when compared to the more common *APOE* ε3 (*APOE3*) allele; whereas the much rarer *APOE* ε2 (*APOE2*) allele has been shown to be protective 1. The inheritance of two copies of *APOE4* increases the chance of developing AD by 12 times compared to the risk of a person with two copies of *APOE3*. Homozygous *APOE4* carriers who develop AD also have a lower average age of clinical onset of 68 years of age compared to an average age of onset of 84 for an individual with two copies of *APOE3*. One copy of *APOE4* increases the chance of AD by three times and lowers the average age of onset to 76 years of age 1. Although mentioned in association with AD most frequently, APOE has also been linked to Parkinson's disease 2, frontotemporal dementia 3 and other neurological diseases (reviewed in 4) as well as linked to lower cognition in nondemented aged individuals 5. The pathways by which APOE impacts the development of AD have been widely studied both *in vitro* and *in vivo*, however, the exact mechanisms have yet to be uncovered.

Much of the work looking at APOE in AD investigates its relationship with Aβ. Early *post mortem* work found a positive correlation between *APOE4* allele dose and Aβ plaque density in individuals with AD 6. A wide range of compelling studies indicate that APOE4 affects the production, clearance and aggregation of Aβ (reviewed in 4, 7). Recent genetic data that strongly suggest inflammation to play a role in AD risk have re‐invigorated the investigations of the role of APOE in neuroinflammation and how this contributes to disease 8; and there are emerging data suggesting that APOE may also influence tau‐mediated neurodegeneration 9.

We hypothesize that APOE4 acts beyond its well‐known roles in influencing Aβ pathology and lipid homoeostasis and has a strong influence on neurodegeneration, inflammation and potentially the spread of pathological proteins through the brain. Here, we conducted a systematic literature search and review the current support in the literature for this hypothesis.

## Methods

**Figure 1:**
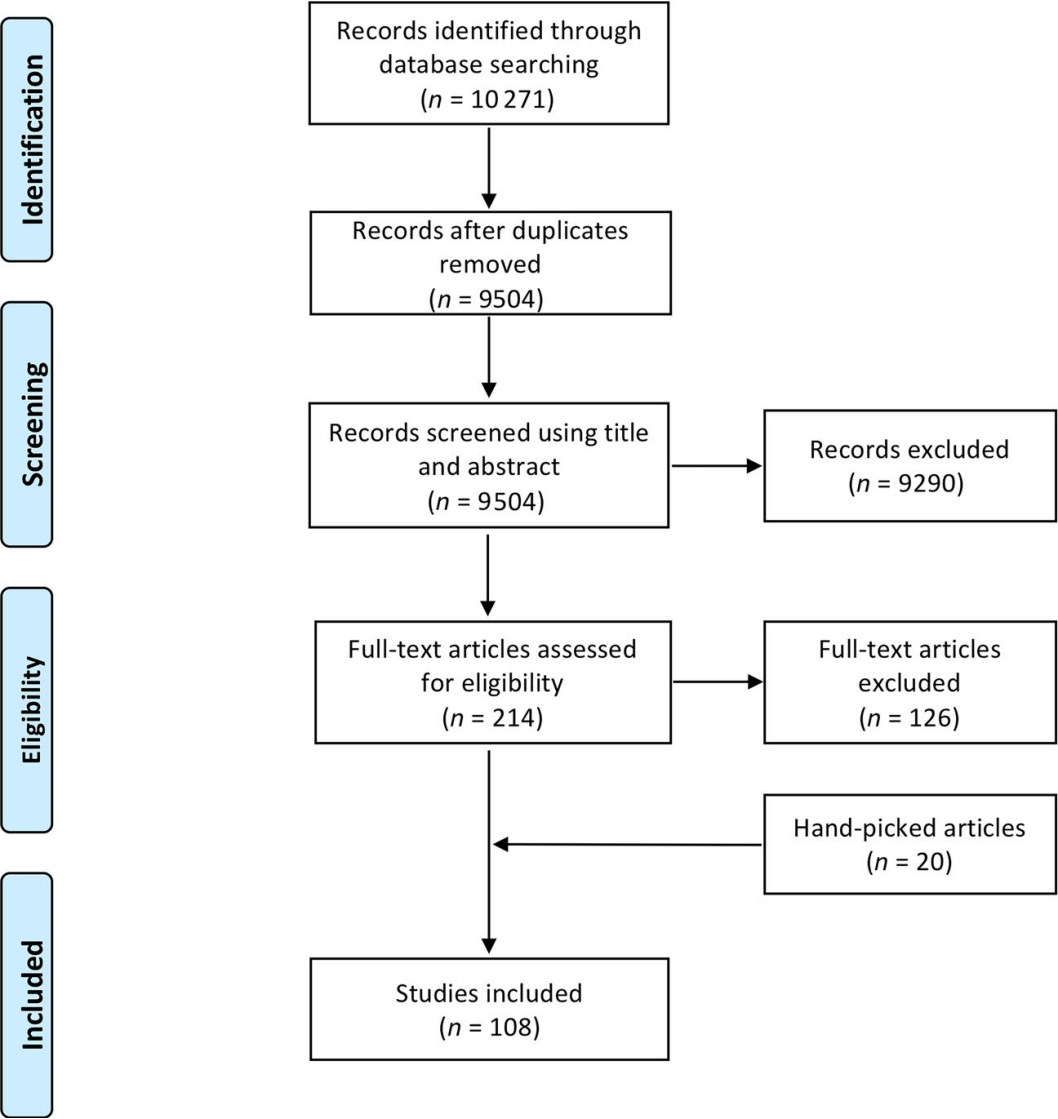
PRISMA flow diagram summarizing the review process. Template edited from 10. Papers referenced in the main body of text were identified through our systematic search and can be found in Table S3.

A systematic literature search approach was taken for finding studies to review in this paper. In February 2018, Embase, Web of Science and MedLine were searched to identify primary research articles published from 1980 to the date searches were run. Search terms covering APOE, AD and inflammation/pathological protein spread/neurodegeneration were developed (Table S1) to suit each database. Initially, there were no language or selection restrictions on the type of study included or how outcomes were defined, measured or when they were taken. Searches identified 22 909 abstracts and titles that were exported to Endnote, where 12 638 duplicates were removed. 10 271 articles were uploaded into Covidence, where a further 767 duplicates were removed. A two‐stage screening strategy was conducted on titles/abstracts and then on full texts using predetermined exclusion criteria (Table S2). Three researchers contributed to the abstract and title screening process such that 50% of abstracts/titles were screened by at least two people and 50% by one. All full‐text articles were double screened. Of the 9502 titles/abstracts screened, 214 progressed to full‐text review and 88 studies were included. Twenty hand‐picked papers that were either published after the search date or were missed during the search but deemed pertinent to the review were also included as is standard practice for full systematic reviews (Figure 1; Table S3). In the results section, we synthesize the findings of the papers identified by the systematic search. Due to the heterogeneous nature of the studies, we did not perform standardized quality control checks of all of the papers, however, all papers included were published in peer‐reviewed journals. Because systematic reviews are designed to test evidence of an intervention and due to the inability to conduct formal quality control due to the many types of experiments reviewed, this is not a fully registered systematic review but instead uses some of the principles of systematic reviews to perform a *systematic literature search* and screen for relevant papers which we review.

## Results of the systematic literature search

### APOE and neurodegeneration

To determine whether there is evidence that APOE influences neurodegeneration search terms were used to identify papers containing both APOE and indicators of neuron and synapse loss (papers relating to APOE and neurodegeneration identified in the systematic search are coloured blue and orange in Table S1).

### APOE‐related atrophy and neuronal loss in AD

Imaging studies in AD populations repeatedly demonstrated possession of an *APOE4* allele to be associated with more extensive atrophy in disease‐specific brain regions such as the medial temporal lobe 3, 11, 12, 13, 14, 15, 16, 17, 18, 19, 20, 21, 22, 23, although this was not universal 24, 25. This is unsurprising when considering that brain structures located here have clinical correlates to well‐defined AD symptoms (e.g. memory impairments – hippocampus; altered emotional responses – amygdala), and that *APOE4* carriers are at increased risk of developing these symptoms in the form of AD. APOE4‐related atrophy of medial temporal lobe structures was suggested to occur in a gene‐dose‐dependent manner 15, 16, 17, 19, 21, with one study finding each *APOE4* allele to impart a 4.8% reduction in hippocampal volume and a 3.8% reduction in amygdala volume 16. In longitudinal studies, accelerated rates of hippocampal atrophy were associated with the *APOE4* allele 13, 22, 26, 27, 28, 29, although this was not always observed 30, 31. APOE4‐related atrophy was also observed in the parietal cortex 3, 13 and some prefrontal areas 3, 12, although again these were not consistently reported and may be related to the inclusion of younger AD patients in these studies.

Diffuse cerebral atrophy is a gross pathological feature of AD. Somewhat surprisingly, however, whole brain volume has been proposed to increase with increasing number of *APOE4* alleles in AD patients 19, 32. Greater *APOE4* allele dose was associated with larger volumes in the frontal lobes 11, 17, 20, although not always 12, 14, which might potentially outweigh reductions in other brain regions and account for increased whole brain volume. Nonetheless, these studies indicate that the *APOE4* allele selectively influences the topography of regional brain atrophy, and thus neurodegeneration, in AD.

Studies of *post mortem* human AD brain have identified neuronal loss in vulnerable brain regions to be characteristic of AD. From our literature search, determining whether APOE influences this was less clear. In the nucleus basalis, APOE4 was found to exacerbate neuronal loss 33, but have no impact in other studies 34, 35. These discrepancies may be due to the different methods employed in quantification of neuronal loss and groups studied. *APOE4* allele possession was also found to enhance neuronal loss in other subcortical structures 33, 34, but have no effect on the CA1 region of the hippocampus or superior temporal sulcus 34, 36.

*In vivo* and *in vitro* studies supported an association between APOE4 and neuronal degeneration. In aged mice expressing human APOE, those expressing APOE4 exhibited increased hippocampal and cortical atrophy compared to those expressing APOE3 37, 38. In APOE4, but not APOE3 mice, activation of the amyloid cascade by inhibition of the Aβ degrading enzyme neprilysin was sufficient to induce degeneration of hippocampal and entorhinal cortex neurons, suggestive of a specific effect of APOE4 in exacerbating Aβ‐related neuronal loss 39. Isoform‐specific effects of APOE on Aβ‐induced neurodegeneration were also suggested *in vitro*, with APOE3 and APOE2, but not APOE4, protecting hippocampal and cortical neurons from Aβ‐induced neurotoxicity 40, 41, 42. APOE4 has also been shown to exacerbate neurodegeneration in the absence of Aβ, instead operating through a tau‐dependent mechanism 9.

Animal studies support an isoform‐specific role for APOE in the loss of GABAergic interneurons 43, 44. Mice expressing human APOE4 exhibited greater age‐dependent loss of GAD67‐ and somatostatin‐positive interneurons in the dentate gyrus compared to those expressing APOE3. Interestingly, loss of somatostatin immunoreactivity was exacerbated by APOE4 in the AD brain 45. The detrimental effects of APOE4 on GABAergic interneurons was sex‐dependent, only being observed in female mice 44. This is interesting when considering APOE4 confers greater AD risk in females and some effects of APOE4 on regional brain atrophy are more prominent in females 16, 18, 21.

In summary, there is strong evidence that APOE4 influences neuron loss in AD, however, there are some conflicting reports and future well‐powered, rigorous studies in both human brain and animal models are needed to fully understand the age, sex and region‐specific effects of APOE on neuron death.

### APOE‐related synaptic and dendritic degeneration in AD

Prior to the onset of neuronal loss in AD, extensive synapse loss and dendritic changes occur. These can be considered early neurodegenerative processes that contribute to synaptic and neuronal dysfunction, which pave the way for more generalized neurodegeneration later in the disease. In contrast to neuronal loss, synaptic and dendritic degeneration are dynamic processes, with the potential to be reversed if targeted early enough. Thus, it is important to consider the role of APOE in these changes.

Whether synapse loss associates with *APOE* genotype was the subject of a few studies identified by our search. Electron microscopy coupled with stereological counting in *post mortem* tissue identified AD‐related loss of synapses in the dentate gyrus, stratum radiatum of the CA1 region of the hippocampus and lamina III of the inferior temporal gyrus. However, this was not related to *APOE* status 46, 47, 48. These findings contrast with results from aged APOE mice, which suggested APOE4 to be associated with a reduced number of synapses in the dentate gyrus 49. Although not accounting for these discrepancies, it is worth noting that electron microscopy is limited by its ability to only quantify synaptic changes in small areas of tissue. Thus, its use for characterizing changes in a disease that stereotypically results in widespread pathology may not be entirely representative.

In AD, loss of the presynaptic vesicle protein, synaptophysin, is evident in various brain regions. In *post mortem* studies, *APOE* genotype did not modulate synaptophysin levels in frontal or temporal regions 50, 51 although a trend towards lower synaptophysin immunoreactivity was observed in AD patients with an *APOE4* allele 50. A further study of another presynaptic vesicle protein, Rab3a, also found no association of synapse loss with *APOE* genotype 52. This contrasts with animal studies demonstrating that mice expressing human APOE4 alone or in concurrence with human amyloid precursor protein display increased age‐dependent degeneration of synaptophysin‐positive presynaptic terminals in the neocortex and hippocampus 53, 54. Preservation of synaptophysin‐positive presynaptic terminals in aged APOE4 mice has also been reported, however, 55. Contradictory results have also been reported in APOE KO mice, with both age‐dependent reductions in synaptophysin‐positive terminals 56 and no changes 57 observed.

Although the findings discussed thus far tend to suggest that APOE4 does not contribute to synaptic degeneration, it is important to consider that techniques used may not be optimal for quantification of synapses or synaptic protein loss, and that many of these studies did not consider plaque proximity in the analyses which is known to drive substantial local synapse loss as will be discussed below. The axial resolution of even confocal microscopy is not sufficient to resolve individual synapses in standard tissue sections. In addition, immunoblotting is inferior to techniques that yield absolute synaptic protein concentrations. Indeed, using ELISA, presynaptic protein levels were found to be reduced in AD patients with an *APOE4* allele, although only a trend towards reduction was seen for synaptophysin 58.

A relatively recently described histological technique, array tomography, has been used to overcome some of the limitations associated with synapse quantification using other methods, offering a means for high‐resolution characterization of synapses in *post mortem* tissue. In addition, this approach avoids some issues associated with electron microscopy in that thousands of synapses can be analysed. Using this technique, synapse density was found to be specifically reduced within the ‘halo' surrounding amyloid plaques. In both AD human *post mortem* tissue and a mouse model, this was isoform‐specific, with APOE4 exacerbating peri‐plaque synapse loss 59, 60.

APOE4‐related peri‐plaque synapse loss affects both pre‐ and post‐synapses 59 and greater age‐induced reductions in post‐synaptic proteins have been observed in AD mouse models expressing APOE4 compared to other isoforms 61. Dendritic abnormalities, such as dystrophic neurites, alterations in dendrite complexity and loss of spines are widespread in AD. Considering that dendritic abnormalities are closely linked to synaptic dysfunction, and thus potentially synaptic degeneration, the effect of APOE here is relevant.

The presence of dystrophic neurites are a neuropathological hallmark of AD and are exacerbated by APOE4 in AD mouse models when compared to other isoforms 59. Such changes in dendritic morphology are predicted to alter synaptic function, and thus may influence degeneration. Indeed, neuritic degeneration requires the presence of APOE 62, with APOE4 mice showing increased age‐dependent loss of neocortical and hippocampal dendrites compared to APOE3 mice 53.

The density of dendritic spines, the post‐synaptic site of over 90% of excitatory neurons, has consistently been shown to be reduced in the presence of APOE4 in cortex, hippocampus, entorhinal cortex and amygdala *in vitro* 63, *in vivo* 64, 65, 66, 67, 68 and in humans 68. A reduction in dendrite length was also observed in APOE4 mice 66, 67, which contributes to reduced connectivity. Interestingly, expression of APOE2 can rescue reduced spine density in AD mouse models 69. The morphology of dendritic spines has also been suggested to be influenced by APOE, with APOE4 being associated with shorter spines and APOE2 with longer spines 64. In addition, APOE may influence spine morphology such that APOE4 specifically reduces the number of spines associated with learning and memory 65. Finally, APOE4 is associated with reductions in dendritic arborization and less complicated branching patterns, impacting on neuronal function 64, 65, 67.

Collectively, these studies suggest that, in the context of dendritic and synaptic changes, APOE4 is less effective at maintaining synaptic and neuronal integrity in disease‐specific brain areas than other isoforms, which likely contributes to synaptic and neuronal degeneration.

### Mechanisms of APOE‐related neurodegeneration

Dendritic, synaptic and neuronal degeneration are all influenced by APOE in an isoform‐specific manner. However, the mechanisms by which APOE4 impacts neurodegeneration are not completely understood. Elucidating these mechanisms may enable the development of appropriate therapies, particularly in relation to APOE effects on synapses and dendrites, which have potential to be reversible.

**Figure 2:**
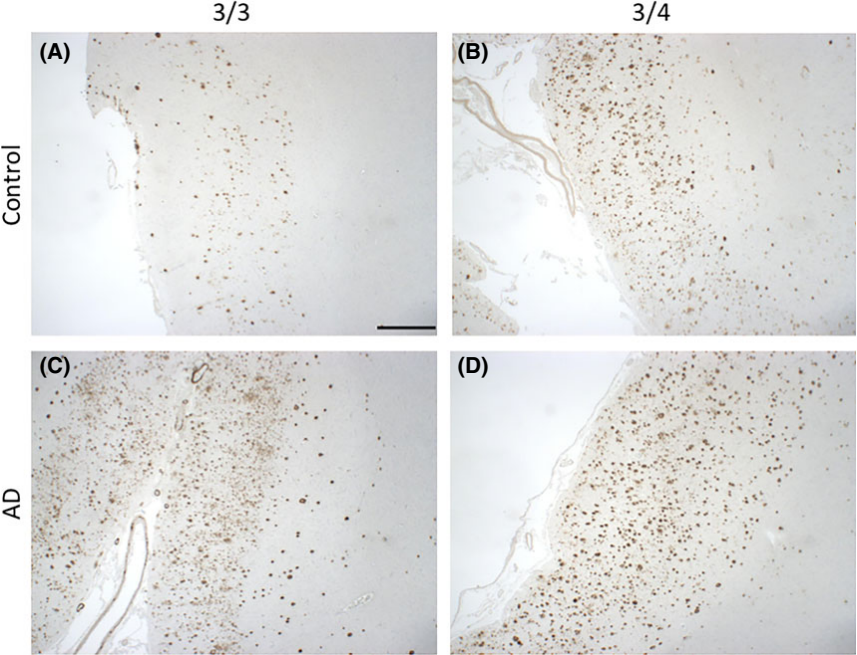
Amyloid‐β pathology in APOE3/3 and APOE3/4 carriers in normal ageing and AD. Aβ plaque deposition is evident in aged controls (**A**) and is exacerbated in the presence of the *APOE4* allele (**B**), resembling an AD‐like phenotype. Both APOE3/3 and APOE3/4 AD cases (**C** and **D**, respectively) have substantial Aβ deposition in all six layers of the cortex. Images taken from the grey matter of inferior temporal lobe (Brodmann area [BA] 20/21). Information about all participants donated tissue can be found in Table S4. Aβ is stained with 6F/3D (mouse monoclonal, DAKO, M087201‐2, 1:100, 98% formic acid, 5 minutes). Scale bar 1 mm.

**Figure 3:**
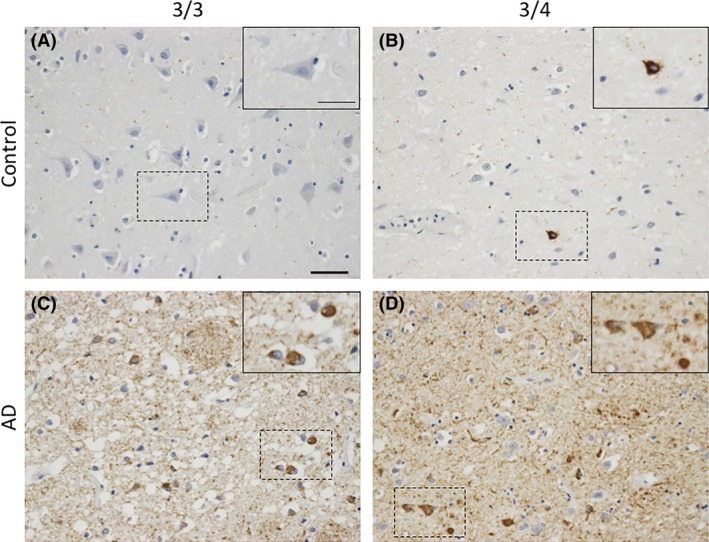
Tau pathology in APOE3/3 and APOE3/4 carriers in normal ageing and AD. In aged controls, phosphorylated tau species are absent in APOE 3/3 cases (**A**) and rarely found in APOE3/4 cases (**B**). In AD, both APOE3/3 (**C**) and APOE3/4 (**D**) have markedly increased numbers of tau‐positive neurones. Images taken from the grey matter of inferior temporal lobe (BA20/21). Phosphorylated tau is stained with AT8 (mouse monoclonal, ThermoFisher, 1020, 1:2500). Scale bar 50 μm, insert scale bar 25 μm.

**Figure 4:**
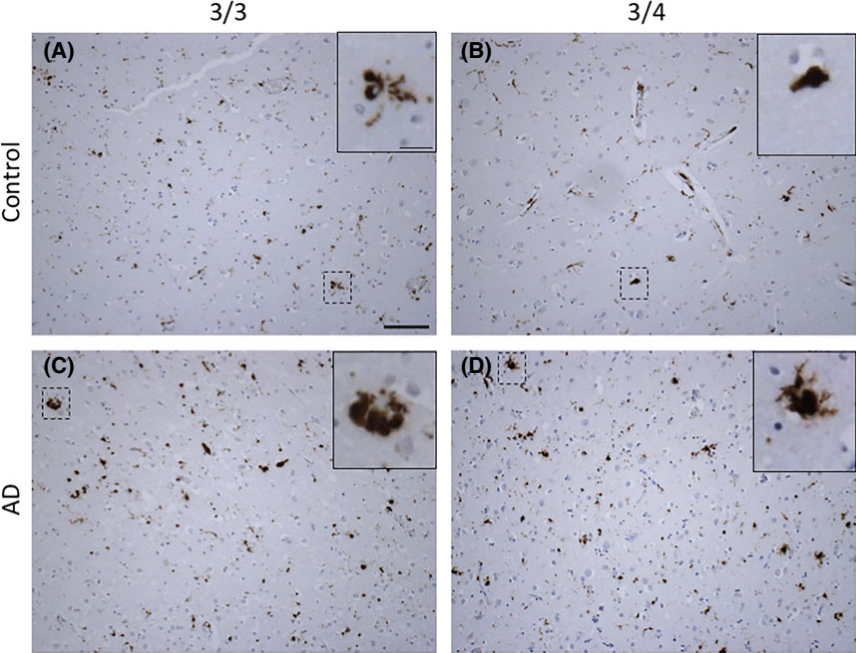
Activated microglia (CD68) in APOE3/3 and APOE3/4 carriers in normal ageing and AD. The lysosomal marker of microglia and macrophages, CD68, shown in AD (C‐D) and age‐matched control cases (**A**–**B**). Various microglial morphologies can be observed, for example, ramified (**A** and **D**) and amoeboid (**B** and **C**) in both ageing/AD and APOE3/x. Images taken from the grey matter of inferior temporal lobe (BA20/21). Microglia are stained with CD68 (mouse monoclonal, DAKO, M0876, 1:100, citric acid antigen retrieval). Scale bar 100 μm, insert scale bar 25 μm.

**Figure 5:**
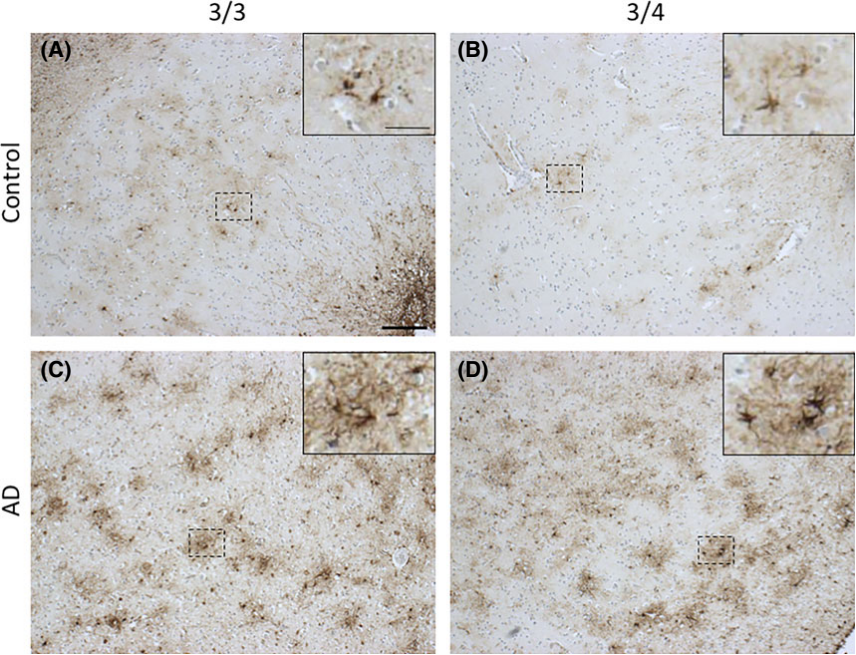
Activated astrocytes in APOE3/3 and APOE3/4 carriers in normal ageing and AD. Glial fibrillary acid protein (GFAP) is a cytoskeletal protein in activated astrocytes. Activated astrocytes are seen in both ageing (**A**–**B**) and AD (**C**‐**D**), but more pronounced astrogliosisis is observed in AD. In AD, astrocytes express more GFAP in the cell bodies, thus appearing darker, especially in APOE3/4 cases (**D**), and have more processes (**C**–**D**). Images taken from the grey matter of inferior temporal lobe (BA20/21). Astrocytes are stained with GFAP (rabbit polyclonal, DAKO, 0334, 1:800). Scale bar 200 μm, insert scale bar 100 μm.

*In vivo* and *in vitro* studies suggested that APOE4 may drive neurodegeneration through an Aβ‐dependent mechanism 37, 39, 40, 41, 42. Recent revisions of the amyloid hypothesis of AD, have suggested soluble oligomeric forms of Aβ (oAβ) to be the effectors of Aβ‐induced degeneration, with APOE4 exacerbating oAβ‐associated degeneration relative to other isoforms 41, 42, 70 (Figure 2) (details of participants in Figures 2, 3, 4, 5 found in Table S4). APOE and oAβ may act intracellularly to enact this degeneration, with APOE uptake into neurons correlating with neuronal death and intracellular accumulation of soluble Aβ 71. APOE4‐specific increases in intraneuronal oAβ have been suggested to drive neurodegeneration through impairments of mitochondria and lysosomes 39, 72, although the role of intracellular Aβ remains hotly debated in the field. Isoform‐specific interactions between APOE and the C‐terminal domain of soluble Aβ, or lack of in the case of APOE4, have also been suggested to influence the propensity for APOE4 to promote Aβ‐mediated neuronal death 42. Moreover, protection against oAβ‐mediated synaptic loss by APOE3 has been suggested to be mediated by a novel intracellular protein kinase C pathway, which is not activated by APOE4 70.

APOE and oAβ may also act extracellularly to impact neurodegeneration. Interestingly, oligomeric forms of Aβ are increased in the ‘halo' surrounding plaques, an area where synaptic degeneration is exacerbated by APOE4 59, 60. Mechanistically, APOE4 might exacerbate peri‐plaque synapse loss by facilitating the association of oAβ with synapses where it is toxic, thus resulting in synapse loss 60.

While APOE certainly influences Aβ‐dependent neurodegeneration, it is becoming increasingly clear that there are other mechanisms by which APOE influences degeneration (Figure 3). For example, in mice expressing a form of mutant human tau associated with frontotemporal dementia, co‐expression of human APOE4 led to a drastic increase in neurodegeneration compared to that seen with other APOE isoforms. APOE was necessary for this tau‐mediated neuronal death to occur 9, suggestive of APOE exerting a neurodegenerative effect through tau. APOE4 has also been shown to impair GABAergic interneurons though a tau‐dependent mechanism *in vivo* 43. Although known to be related to Aβ, dysregulation of calcium homoeostasis has also been implicated as a mechanism of APOE‐related neurodegeneration, independent of Aβ. Here, APOE4 increased levels of cytosolic calcium, an effect that was dose‐dependently associated with cell death 73.

Another important consideration, and one that is often neglected, is the cellular source of APOE. Although primarily produced by glial cells, under conditions of stress or injury neurons also synthesize APOE 74. Considering that the AD brain can be considered both a ‘stressed' and ‘injured' environment, the cellular source of APOE is likely to be relevant to its effect on neurodegeneration. Indeed, at the level of the dendrite, loss of spines, reduced arborization and alterations in morphology were observed in mice expressing neuronal APOE4, but not astrocytic APOE4 65, 75. This phenomenon has also been confirmed to occur at the level of the synapse, with only neuronal APOE4 promoting the degeneration of presynaptic terminals and cell death 75. Consequently, the cellular source of APOE seems to impact upon its capacity to induce neurodegeneration. Further studies are needed to examine how neuronal and astrocytic APOE differ from one another, to characterize how these effects are mediated.

A potential mechanism by which neuronal APOE4 exerts increased neurotoxic effects may be due to the intraneuronal proteolytic processing of APOE, whereby APOE can be cleaved to generate C‐terminally truncated fragments. In the human AD brain, these fragments are more numerous in individuals carrying an *APOE4* allele. *In vitro*, APOE4 is more susceptible to proteolytic cleavage than APOE3 and, in mice, these APOE fragments are capable of eliciting AD‐like neurodegeneration 76. Further work has identified the lipid‐binding region of APOE to be essential for toxicity although not sufficient alone. Instead, the lipid‐ and receptor‐binding regions appear to act in concert to mediate toxic effects 77. APOE4 fragments have also been shown to interact synergistically with Aβ and tau, exacerbating both pathologies and increasing the degree of neurodegeneration 43, 78. In relation to Aβ, APOE4 fragments bind poorly to Aβ, leading to reduced clearance and increased deposition 78, and these fragments also promote intraneuronal Aβ accumulation 79. In relation to tau, APOE fragments increase the degree of phosphorylation, likely exacerbating neurodegeneration 43. Cleavage fragments of APOE may also exert neurodegenerative effects by increasing intracellular calcium 80, a mechanism that has previously been associated with full‐length APOE4, as aforementioned, or by impairing mitochondrial function and integrity 77. Finally, structural differences among APOE isoforms may also contribute to the more neurodegenerative phenotype associated with APOE4. Unlike APOE2 and APOE3, APOE4 exhibits a domain interaction, whereby a salt bridge mediates interaction between N‐ and C‐terminal domains. Indeed, in mice, this domain interaction has been shown to be associated with pre‐ and post‐synaptic protein loss 81, thus suggesting another mechanism by which APOE4 might contribute to synaptic pathology and neurodegeneration.

APOE genotype may further contribute to synaptic degeneration, and subsequent neuronal degeneration, through its effects on dendrites. Dendritic changes are suggested to alter neuronal plasticity and regenerative capacity, leading to synaptic dysfunction and subsequent synapse and neuron loss. Indeed, more plastic changes are seen in the human AD brain in the absence of an *APOE4* allele 33. Various mechanisms have been proposed as to how APOE isoforms differentially regulate dendritic changes in AD. These range from altered binding of APOE to receptors and subsequent intracellular signalling cascades 82, 83, impaired regulation of receptors within spines 63, elevations in calcineurin activity that is associated with reductions in spine density 84 and impairments of neuronal outgrowth 82, among others.

Overall, these results highlight the far‐reaching and diverse biological effect APOE4 has on neurodegeneration in AD with particularly strong evidence supporting a role for APOE4 in synapse degeneration.

### APOE and inflammation

To determine whether there is evidence that APOE influences inflammation, search terms were used to identify papers containing both APOE and indicators of inflammation (papers from the systematic search that support a role for APOE in inflammation are coloured in green and orange in Table S1).

### Increased glial activation and gliosis with APOE4

There is substantial reactive glial cell accumulation, termed gliosis, during AD which is enhanced in the presence of the *APOE4* allele (Figures 4 and 5). Markers of glial activation are commonly used to reflect a variety of functional outcomes and to quantify the changes in glial numbers and their respective phenotype.

Human *post mortem* studies have quantified gliosis in different brain regions using multiple markers of activation. By immunophenotyping microglia in the frontal gyrus and correlating to APOE status in AD and control cases, markers of activation (CD68, Human Leucocyte Antigen‐DR isotype [HLA‐DR], CD64) were found to be significantly associated with APOE4 carriers while APOE2 carriers were associated with higher levels of more homoeostatic microglial markers (Iba1 and Macrophage Scavenger Receptor‐A; MSR‐A) and lower levels of the reactive ones 85. Although the microglial phenotype was insufficient in predicting the APOE status in dementia, the elevated markers of phagocytosis, adaptive immune response and antigen recognition seen in APOE4 carriers point towards a more pathological environment. Other brain regions have been assessed for microglial levels, where in an *APOE*4 dose‐dependent manner, there was extensive microgliosis in both the frontal and temporal cortices 86. Similarly, there is elevated GFAP‐positive astrogliosis in the grey matter of APOE4 AD patients compared to other *APOE* genotypes, and an overall greater astrogliosis between AD and age‐matched control cases 87. Interestingly, the *APOE4* allele was associated with differences in GFAP burden in nondemented individuals, despite the increased plaque burden of nondemented aged APOE4 individuals, supporting the importance of glial cells in AD pathogenesis. As always, the *post mortem* tissue only provides a snap‐shot of end‐stage of disease, so we rely on mouse models for deciphering the mechanistic changes that APOE4 induces during AD.

Mouse studies looking at the effects of the human *APOE* allele with AD‐like pathology have mostly replicated the gliosis data seen in human *post mortem* tissue. APOE4 mice crossed to the 5xFAD amyloidosis model showed increased microgliosis in deep cortical layers accompanied by a greater number of dystrophic microglial processes in the presence of the *APOE4* allele, compared to *APOE4* and *APOE3* 88. Of note, both APOE2 and APOE4 5xFAD mice had more Aβ‐associated microglia than APOE3 88, 89, suggesting that the APOE2 conformation may be protective not by preventing microglia/plaque interactions but by mediating more effective ways to respond to the plaques and making microglia more resistant to amyloid toxicity. These changes were not observed in the subiculum of these mice, reiterating regional differences in microglia. Furthermore, when APOE knock‐in mice were crossed to a tauopathy model (P301S), CD68‐positive microglial burdens in the hippocampus and entorhinal/piriform cortex were markedly increased in APOE4 mice compared APOE3 and APOE knock‐out mice in a tau pathology mediated manner 9. The same effect was also observed with GFAP immunoreactivity, further establishing an aberrant glial response to AD‐like pathology in combination to the presence of the *APOE4* allele. Similarly, there was neurodegeneration‐associated microgliosis and astrogliosis in the hippocampus of APOE4 mice, compared to APOE3, but no differences were found in the septum 90. However, in the hippocampus of APOE4 LPS‐injected mice there was marked microgliosis although astrogliosis was found in the APOE3 mice 91. In summary, in APOE4 amyloidosis (5xFAD) and tauopathy (P301S) mouse models there was exacerbated gliosis. Although, there was important regional variability, taken together these data strongly support a role for APOE4 in promoting inflammatory changes in microglia and astrocytes.

The precise mechanisms by which reactive gliosis is established in the APOE4 AD brain is unknown, but a key question that remains is whether this gliosis is a driver of the disease, accounting for the earlier onset and worse prognosis *APOE4* carriers face, or a by‐product of the exacerbated amyloid and tau pathology. To answer these questions, the inflammatory capacity of glial cells and their transcriptomic signatures in the presence of the APOE4 are being currently assessed.

### *Apoe*‐related glial transcriptional changes

Transcriptional studies are becoming increasingly popular in the microglia and neuroinflammation field, with *Apoe* upregulation consistently being a top hit in AD‐like mouse models. The advantages of RNA‐sequencing are a nonbiased, high‐yield output of all transcriptional changes as well as the ability to use these long data sets to investigate biological pathways. As such, this process speeds up the identification of genetic and molecular pathways involved in AD pathogenesis and ways they can be therapeutically targeted.

Recently, microglial *Apoe* mRNA transcript levels were quantified in two models of AD‐like pathology (amyloidosis and tauopathy) and ageing 92. Its high abundance in all conditions supports a role for APOE as a key part of the microglial signature, despite its expression not being restricted to microglial cells. In terms of its relative expression, *Apoe* was highly upregulated in ageing and disease models, with ageing female mice showing a marked increase. Pathway analysis puts APOE as the driver of a network whose downstream effectors are also highly upregulated in these models, like the chemoattractant CCL3, whose relevance to neuroinflammation will be discussed in the next section of this review.

*Apoe* transcription is also downstream of the activation of a microglial receptor TREM2 (Triggering Receptor Expressed on Myeloid cells 2), another AD risk gene 93. This APOE activation pathway results in a more pro‐inflammatory microglial response and a degenerative phenotype, as seen in the AD brain. Microglial and astrocytic pro‐inflammatory genes were also profoundly upregulated in APOE4 knock‐in mice crossed with a tauopathy model (P301S), compared to APOE3 9. Conversely, APOE knock‐out/P301S mice showed attenuation of this impaired pro‐inflammatory profile, highlighting APOE as a master regulator of glial inflammatory response with its *APOE4* allele being associated to a pro‐degeneration phenotype.

Single‐cell RNA sequencing takes this a step further by identifying and characterizing clusters of subpopulations within a cell type, for instance disease‐associated microglia (DAMs) 94. Specifically, *Apoe* is upregulated early in DAMs of the 5xFAD AD‐like model, even in the absence of TREM2. A TREM2 independent pathway is thus proposed to initiate Apoe upregulation in the early phase of AD, with a later TREM2‐dependent pathway activating Apoe transcription which induces neurodegenerative microglia. This potentially forms a therapeutic window where preventing the second Apoe induction via TREM2 may protect against the exacerbated inflammation and degeneration caused by microglia. Understanding the ways in which TREM2 is activated in the AD brain and the effectors mediating this TREM2‐APOE pathway can provide new ways to halt AD progression and hinder neuroinflammation.

Human induced pluripotent stem cells (iPSCs) derived from AD patients were recently transcriptionally characterized after directing them towards a microglial‐like lineage 95. Importantly, the microglia‐like cells were engineered with Crispr‐Cas9 to correct APOE4 into APOE3. Not only did this result in attenuation of AD‐associated morphological and transcriptomic signatures, but immune‐related genes were also upregulated.

Overall, *Apoe* is consistently one of the most upregulated genes in transcriptomic studies of microglia in AD‐like models. Nevertheless, a limitation of transcriptomics involves the isolation process which pushes microglia into an activated phenotype 92. There is a lack of detailed studies of human *post mortem* tissue profiling transcription in cases with different APOE genotypes, which will be needed to confirm the translational relevance of mouse studies. As this is still at the transcriptional level, studies looking a more functional level are imperative to understand how these RNA changes relate to disease.

### Altered inflammatory response by glial cytokine release

Innate and adaptive immune cells respond to environmental stimuli by releasing signalling cytokines and chemokines. Our systematic literature search showed that the process of cytokine release by glia to maintain homoeostasis and respond to damage is dysfunctional in the ageing and AD brain 96, with substantial evidence pointing towards the *APOE4* allele playing a crucial role in this 97, 98, 99, supporting the transcriptional profile changes seen in ε4 microglia and astrocytes.

APOE4 alters the baseline pro‐inflammatory response even in the absence of disease. Addition of APOE4, but not APOE3 protein, to rat microglia cultures alone stimulated the secretion of prostaglandin E2 (PGE2), interleukin‐1β (IL‐1β), and nitric oxide (NO) 100, 101, 102, 103. On the other hand, microglial and astrocyte stimulation with APOE4 and Aβ attenuated the production of inflammatory mediators 103, 104, 105, indicating a more complex interaction of microglia, APOE, and Aβ *in vivo*. A physiological concentration of Aβ may therefore be beneficial to glial functioning by interacting with APOE.

LPS‐activated microglia and astrocytes are a well‐characterized model of glial activation by mimicking the inflammation seen in AD. Microglia induced with LPS in the APOE4 background released greater amounts of pro‐inflammatory cytokines, like tumour necrosis factor‐α (TNF‐α), IL‐1β, and interleukin‐6 (IL‐6) 106, an effect replicated in AD‐like models 88, 107. Simultaneously, APOE4 treated LPS‐induced microglia suppressed the production of TNF‐α less than the APOE3 and APOE2 isoforms 108 while APOE‐/‐ mice secrete lower levels of anti‐inflammatory cytokines 109, showing a physiological role of APOE in modulating inflammation. Indeed, knocking out murine *Apoe* increases glial production of nitric oxide (NO) 110 and other inflammatory mediators, like *TNF‐*α, *IL‐1*β and *IL‐6* transcripts in the CNS 111. Murine *Apoe* is therefore required to suppress glial‐mediated inflammation providing a physiological role in CNS homoeostasis, which is disrupted in AD, potentially in an age‐dependent manner. This evidence points to APOE4 expressing microglia being both more pro‐inflammatory and less anti‐inflammatory at the same time.

In contrast to the microglial data, astrocytes from APOE2 and APOE3 animals treated with LPS produced more of these pro‐inflammatory cytokines than the APOE4 counterparts (IL‐1β, TNF‐α, and IL‐6) 112. APOE4 astrocytes also produce more CCL3 (chemokine C‐C motif ligand 3) 113, similar to microglial *Ccl3* mRNA upregulation in AD‐like models 92, which is downstream of the APOE‐driven network while APOE‐/‐ mice produce less CCL3 109. Despite the increase in CCL3, the chemoattraction ability of microglia is impaired in the presence of the *APOE4* allele, as they are less migratory and receptive to immune sensing 114, 115, 116. Data so far suggest that APOE4 confers a more pro‐inflammatory and less anti‐inflammatory phenotype in microglia, with an opposing pattern in astrocytes.

### APOE at the interface of inflammation and neurodegeneration: glial‐mediated synapse loss

**Figure 6:**
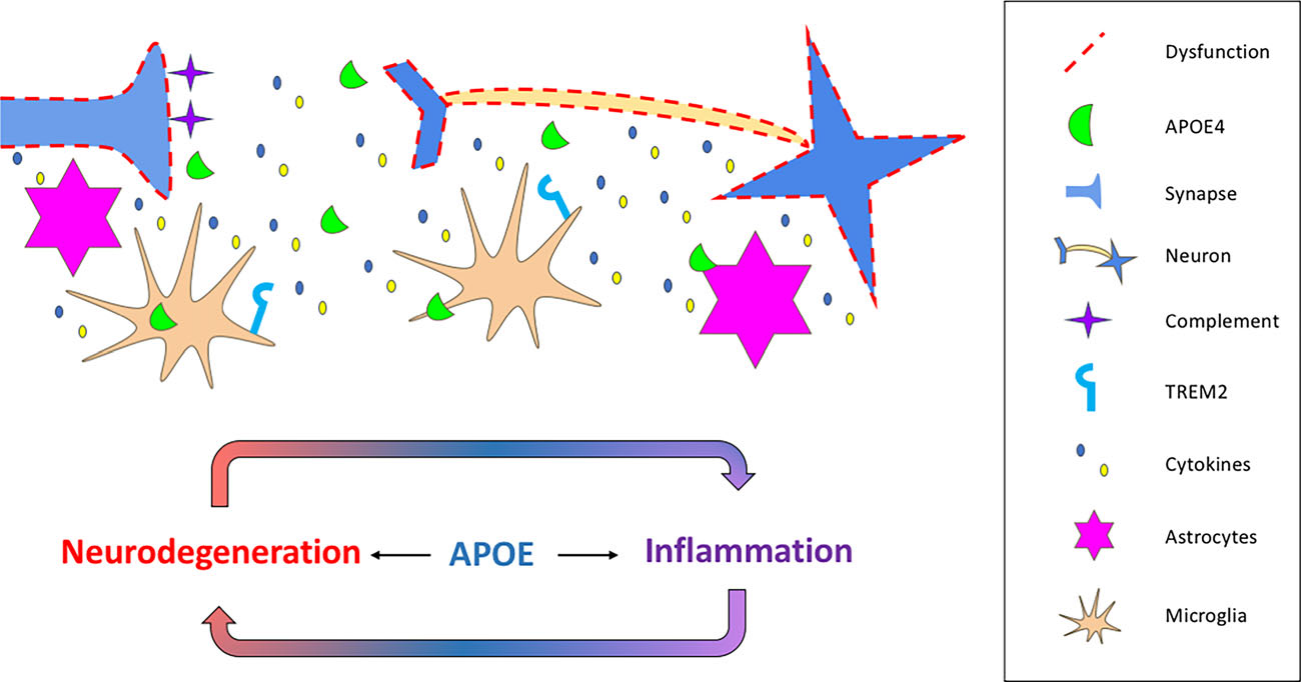
APOE at the interface of inflammation and neurodegeneration via glial‐mediated mechanisms. Microglia and astrocytes expressing APOE4 promote parenchymal gliosis and release pro‐inflammatory signals that are potentially associated with synaptic and neuronal loss. The paracrine signalling of microglial mediators along with the APOE‐TREM2 pathway induces a pro‐inflammatory phenotype, creating a vicious‐cycle of inflammation and neurodegeneration. Ineffective clearance of excess synapses by astrocytes in APOE4 mice allows accumulating levels of C1q that can act as a tag for synaptic elimination later in life.

The synaptic loss during the early phase of AD is now thought to be partly due to aberrant microglial and astrocyte complement‐mediated phagocytosis 117, 118. Given APOE's role in synapse loss and inflammation, we postulate that the APOE4 genotype is implicated in synaptic loss through a glial‐mediated mechanism (Figure 6).

LPS intracerebral injections in APOE4 mice led to decreased pre‐ and post‐synaptic protein levels as well hippocampal gliosis and pro‐inflammatory cytokine release 119. Moreover, APOE4 is accompanied by greater complement activation 120, which is the proposed synaptic tag for synaptic clearance. Still, this evidence is correlative and there are other mediators than could affect synaptic loss. In development, astrocytes of the APOE4 background were less phagocytic towards pHrodo‐labelled synaptosomes than those of APOE3 and APOE2 121, leading to the hypothesis that some synapses are not pruned by APOE4 astrocytes, accumulating complement and making them more vulnerable in AD. Whether these synapses are defective or not is a key question, as loss of healthy synapses with accumulated complement would explain the initial synapse loss in AD, and the earlier onset of APOE4 carriers.

Although a lot more evidence is required, particularly from the human perspective, to understand if and how glial cells drive the synapse loss during AD, understanding why APOE e4 carriers at greatest risk have this extensive synapse loss and greater onset will be crucial to tailor therapies for individuals of this genotype.

### Potential role for spread of pathological proteins through the brain

**Figure 7:**
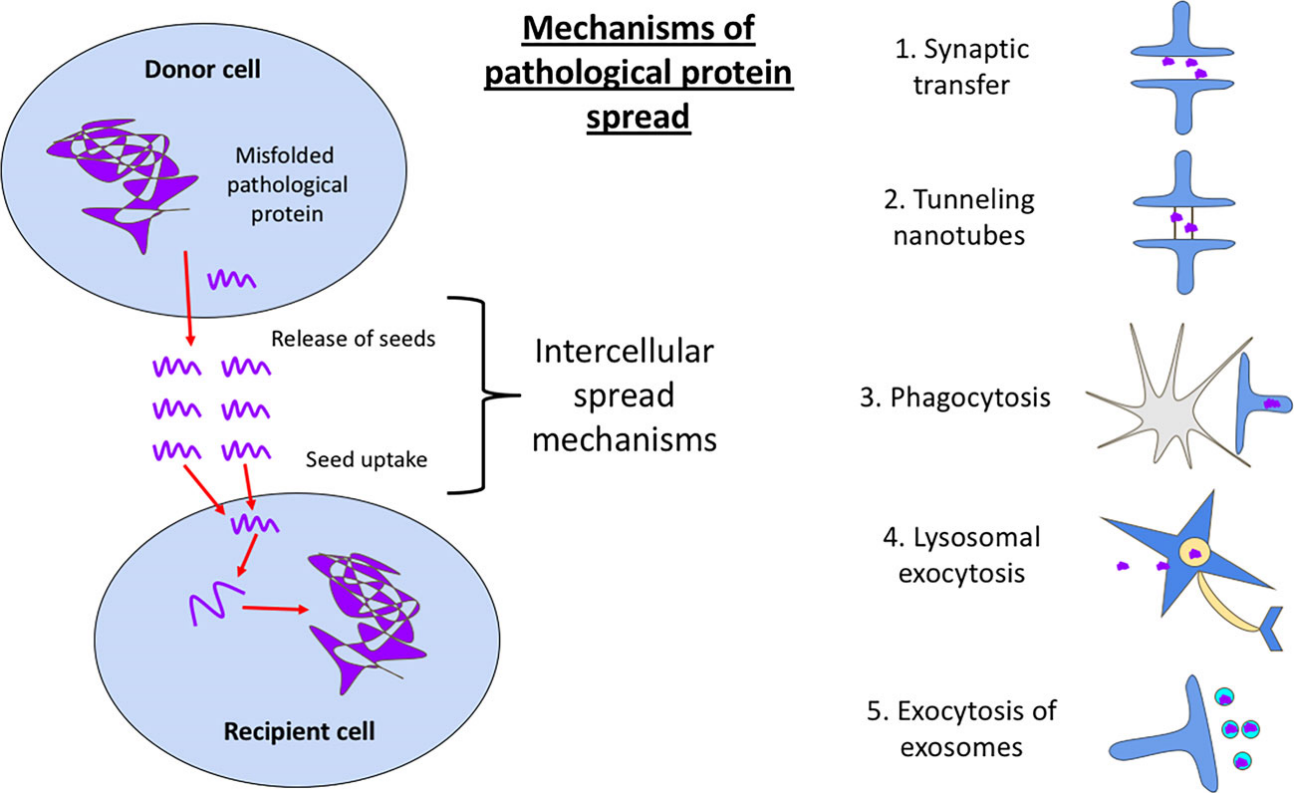
Schematic diagram of pathological protein spread. While direct evidence for APOE influencing the spread of tau through the brain is lacking, the papers in our systematic literature search implicate APOE in many processes that could influence spread. These include synaptic transfer through the synaptic cleft (1), via nanotubes (2), by glial phagocytosis (3) or vesicular secretion (4 & 5).

No studies were found in the systematic search that specifically investigated the role of APOE in pathological protein spread. Two of the current hypotheses about the spread of tau through the brain are that tau spreads trans‐synaptically and that microglia eat tau‐containing synapses facilitating its spread (Figure 7). Interestingly, in this review, APOE genotype is shown to affect both tau and microglia in multiple ways, implicating APOE4 as a potential facilitator of misfolded proteins spreading between brain regions.

## Conclusion

Our systematic literature search revealed strong links between APOE and synapse degeneration, which is further supported by relevant literature that did not fall into our search terms. The impaired phagocytic capacity of TREM‐/‐ microglia for synapses and synaptosomes in development 122 highlights yet again the TREM2/APOE pathway as a potential AD‐related mechanism of synaptic elimination. The interplay between glia cell types in relation to APOE genotype is also a new avenue to be assessed considering that astrocytic IL‐33 induces microglial synaptic engulfment of both excitatory and inhibitory synapses 123. Conversely, microglia have been shown to induce a neurotoxic astrocyte phenotype via IL‐1α, C1q, and TNF 124, the two latter being increased with APOE4 expression. Moreover, APOE4‐elevated soluble factors released by microglia like NO and IL‐6 can induce synapse loss in neurone culture systems 125, suggesting microglia may alter synaptic numbers both by phagocytosis and their secretome.

In studies identified by our systematic search, APOE4 was consistently associated with increased neurodegeneration within the medial temporal lobe; this region includes the entorhinal cortex, where tau pathology begins 126. APOE4‐associated degenerating synapses may release tau seeds via exosomes, stress‐produced nanotubes 127, or passively as terminals degenerate. These may be taken up by recipient cells therefore propagating tau trans‐synaptically. However, our recent data indicate that presynaptic terminal degeneration is not necessary for the spread of tau through neural circuits 128, making it important to investigate other mechanisms of pathological protein spread.

Microglia have also been suggested to mediate tau spread 129. The exacerbated neurodegeneration in APOE4 likely induces microglia and astrocytes to phagocytose degenerating tau‐containing synapses and neurones. Exosomes synthesized from these microglia, containing synaptic‐impairing micro‐RNA 130 and tau seeds, may propagate tau pathology and neurodegeneration 131. Astrocytes may also be involved in this, given their role in α‐synuclein transfer via tunnelling nanotubes in Parkinson's disease 132.

APOE, therefore, is potentially at the interface of inflammation, neurodegeneration, and the pathological protein spread (summarized in Figures 6 and 7). Tau pathology and synapse loss are the strongest correlates with cognitive impairments 133; therefore, preventing these processes could have significant impact on disease progression. Further evidence is, however, needed to directly link APOE to these spreading mechanisms.

Our systematic literature search and review of the resulting papers highlights the need for multiple approaches to understand the complex role of APOE in disease, as has been observed recently for many fields in science 134. Many findings remain contradictory and will need further support and investigation using different model systems to warrant moving forward to therapies targeting APOE. These caveats notwithstanding, our review of the literature supports the idea that understanding how APOE influences multiple pathological features of AD will be important for developing effective therapeutics to prevent or treat the disease. Currently, there is debate as to whether lowering or increasing levels of APOE will be beneficial in treating AD. Increasing astrocytic APOE levels can help displace synaptic Aß 135 and prevent subsequent synaptotoxicity. Such interventions, with the recent example of Bexarotene, have had mixed outcomes in mouse models of AD 136, 137, 138, 139 and no direct benefits so far in human trials 140. In contrast, lowering levels of APOE4 and increasing levels of APOE2 59 and APOE3 141, or decreasing total APOE 142 are promising alternative avenues for maintaining brain resilience and synaptic integrity.

## Author contributions

MT – design of systematic search, paper screening, writing paper, staining and imaging tissue, revising paper. CD – design of systematic search, paper screening, writing paper, revising paper. AN – design of systematic search, paper screening, writing paper. RJJ – writing paper, revising paper. TS‐J – design of systematic search, writing paper, revising paper.

## Ethics

Use of human tissue for *post mortem* studies has been reviewed and approved by the Edinburgh Brain Bank ethics committee and the ACCORD medical research ethics committee, AMREC (approval number 15‐HV‐016; ACCORD is the Academic and Clinical Central Office for Research and Development, a joint office of the University of Edinburgh and NHS Lothian). The Edinburgh Brain Bank is a Medical Research Council funded facility with research ethics committee (REC) approval (11/ES/0022). Tissue from four donors was used for this study and their details are found in supplementary information.

## Disclosures

Authors declare no conflicts of interest.

## Supporting information


**Table S1.** Search terms used for Embase, Web of Science, and Med Science

**Table S2.** Exclusion Criteria

**Table S3**. Papers included in the systematic review (Discussed in the Results sections) (* denotes hand‐picked papers included in the systematic literature search)

**Table S4**. Details of human *post mortem* cases used for Figures 2‐5. MRC BBN: Medical Research Council Brain Bank Number, AD: Alzheimer's disease, PM: *post mortem*.
