# Supplementary material for: Invited Review: APOE at the interface of inflammation, neurodegeneration and pathological protein spread in Alzheimer's disease
Source: Neuropathol Appl Neurobiol. 2018 Nov 28;45(4):327–46. doi: 10.1111/nan.12529 (PMC6563457; doi:10.1111/nan.12529)
Supplement: Supplementary file 1 — Table S1. Search terms used for Embase, Web of Science, and Med Science [file NAN-45-327-s001.docx]

**Supplemental Information 1:** Search terms used for Embase, Web of Science, and Med Science

**Database: Med Science**

**Inflammation**
1. exp Apolipoproteins E/
2. Alzheimer Disease/
3. 1 and 2
4. Astrocytes/
5. Microglia/
6. microglia*.mp.
7. compound granular corpuscle.mp.
8. glitter cell.mp.
9. hortega cell.mp.
10. glia cell.mp.
11. 5 or 6 or 7 or 8 or 9 or 10
12. reactiv*.mp.
13. 4 and 12
14. 11 and 12
15. activ*.mp.
16. 4 and 15
17. 11 and 15
18. exp Complement C4/
19. Complement C1q/
20. Complement C3/
21. Interleukin-1beta/
22. Tumor Necrosis Factor-alpha/
23. cytokine*.mp.
24. GLIOSIS/
25. pro inflammatory.mp.
26. anti inflammatory.mp.
27. Inflammat* mediat*.mp.
28. neuroinflammatory.mp.
29. neuroinflammation.mp.
30. phagocyt*.mp.
31. immun*.mp.
32. 4 or 11 or 13 or 14 or 16 or 17 or 18 or 19 or 20 or 21 or 22 or 23 or 24 or 25 or 26 or 27 or 28 or 29 or 30 or 31
33. 3 and 32

**Neurodegeneration**
1. exp Apolipoproteins E/
2. Alzheimer Disease/
3. 1 and 2
4. neuron*.mp.
5. synap*.mp.
6. 4 or 5
7. dysfunction*.mp.
8. degenerate*.mp.
9. degeneration.mp.
10. Death.mp.
11. Impair*.mp.
12. Loss.mp.
13. dysregulate*.mp.
14. dysregulation*.mp.
15. prun*.mp.
16. phagocyt*.mp.
17. 7 or 8 or 9 or 10 or 11 or 12 or 13 or 14 or 15 or 16
18. 6 and 17
19. neurodegenerat*.mp.
20. Neurotoxic*.mp.
21. synaptotoxic*.mp.
22. Myelin*.mp.
23. Demyelin*.mp.
24. 19 or 20 or 21 or 22 or 23
25. 18 or 24
26. 3 and 25

**Prion-like spread**
1. exp Apolipoproteins E/
2. Alzheimer Disease/
3. 1 and 2
4. Amyloid/
5. Amyloid beta-Peptides/
6. AMYLOID BETA-PROTEIN PRECURSOR/
7. Plaque, Amyloid/
8. tau Proteins/
9. tau.mp.
10. phf.mp.
11. tangles.mp.
12. Neurofibrillary Tangles/
13. paired helical filament.mp.
14. straight filament.mp.
15. tauopathy.mp.
16. neurofibrill*.mp.
17. 4 or 5 or 6 or 7 or 8 or 9 or 10 or 11 or 12 or 13 or 14 or 15 or 16
18. prion*.mp.
19. move*.mp.
20. moving.mp.
21. dynamic*.mp.
22. Propagat*.mp.
23. attenuat*.mp.
24. spread*.mp.
25. secret*.mp.
26. transfer*.mp.
27. transmiss*.mp.
28. progress*.mp.
29. Trans-synaptic*.mp.
30. seed*.mp.
31. infect*.mp.
32. tunnel*.mp.
33. conver*.mp.
34. aggregat*.mp.
35. 18 or 19 or 20 or 21 or 22 or 23 or 24 or 25 or 26 or 27 or 28 or 29 or 30 or 31 or 32 or 33 or
36. 3 and 17 and 35

**Database: Embase**

**Inflammation**
1. apolipoprotein E/
2. APOE.mp.
3. APO-E.mp.
4. apolipoprotein e isoproteins.mp.
5. apoe isoproteins.mp.
6. apo e isoproteins.mp.
7. Apoproteins E.mp.
8. APOE-epsilon2.mp.
9. APOE epsilon2.mp.
10. ApoE2.mp.
11. APOE-epsilon 2.mp.
12. APOE epsilon 2.mp.
13. Apo E-2.mp.
14. Apo E 2.mp.
15. APO E2.mp.
16. Apolipoprotein-epsilon2.mp.
17. Apolipoprotein epsilon2.mp.
18. Apolipoprotein E-2.mp.
19. Apolipoprotein E 2.mp.
20. apolipoprotein E2.mp.
21. APOE-epsilon3.mp.
22. APOE epsilon3.mp.
23. ApoE3.mp.
24. APOE-epsilon 3.mp.
25. APOE epsilon 3.mp.
26. APO E-3.mp.
27. Apo E 3.mp.
28. APO E3.mp.
29. Apolipoprotein-epsilon3.mp.
30. Apolipoprotein epsilon3.mp.
31. Apolipoprotein E-3.mp.
32. Apolipoprotein E 3.mp.
33. Apolipoprotein E3.mp.
34. apoprotein E3.mp.
35. APOE-epsilon4.mp.
36. APOE epsilon4.mp.
37. APOE4.mp.
38. APOE-epsilon 4.mp.
39. APOE epsilon 4.mp.
40. Apo E-4.mp.
41. Apo E 4.mp.
42. apo e4.mp.
43. Apolipoprotein-epsilon4.mp.
44. Apolipoprotein epsilon4.mp.
45. Apolipoprotein E-4.mp.
46. Apolipoprotein E 4.mp.
47. Apolipoprotein E4.mp.
48. 1 or 2 or 3 or 4 or 5 or 6 or 7 or 8 or 9 or 10 or 11 or 12 or 13 or 14 or 15 or 16 or 17 or 18 or 19 or 20 or 21 or 22 or 23 or 24 or 25 or 26 or 27 or 28 or 29 or 30 or 31 or 32 or 33 or 34 or 35 or 36 or 37 or 38 or 39 or 40 or 41 or 42 or 43 or 44 or 45 or 46 or 47
49. Alzheimer disease.mp.
50. Alzheimer.mp.
51. Alzheimer's.mp.
52. Alzheimer Dementia.mp.
53. Dementia Alzheimer.mp.
54. Alzheimer-Type Dementia.mp.
55. Dementia Alzheimer-Type.mp.
56. Primary Senile Degenerative Dementia.mp.
57. Dementia Senile.mp.
58. senile dementia.mp.
59. Dementia Alzheimer Type.mp.
60. Alzheimer Type Dementia.mp.
61. Senile Dementia Alzheimer Type.mp.
62. Alzheimer Type Senile Dementia.mp.
63. Dementia Primary Senile Degenerative.mp.
64. Disease Alzheimer.mp.
65. Alzheimer Disease Late Onset.mp.
66. Focal Onset Alzheimer's Disease.mp.
67. Familial Alzheimer Disease.mp.
68. Sporadic Alzheimer disease.mp.
69. Alzheimer Disease Early Onset.mp.
70. Early Onset Alzheimer Disease.mp.
71. alzeimer disease.mp.
72. 49 or 50 or 51 or 52 or 53 or 54 or 55 or 56 or 57 or 58 or 59 or 60 or 61 or 62 or 63 or 64 or 65 or 66 or 67 or 68 or 69 or 70 or 71
73. astrocyte/
74. microglia/
75. 73 or 74
76. reactiv*.mp.
77. activ*.mp.
78. 75 and 76
79. 75 and 77
80. complement component C4/
81. complement component C1q/
82. complement component C3/
83. interleukin 1beta/
84. tumor necrosis factor/
85. cytokine*.mp.
86. gliosis.mp.
87. pro-inflammat*.mp.
88. anti-inflammat*.mp.
89. Inflammat* mediat*.mp.
90. neuroinflammat*.mp.
91. phagocyt*.mp.
92. immun*.mp.
93. 80 or 81 or 82 or 83 or 84 or 85 or 86 or 87 or 88 or 89 or 90 or 91 or 92
94. 73 or 74 or 78 or 79 or 93
95. 48 and 72 and 94

**Neurodegeneration**
1. apolipoprotein E/
2. APOE.mp.
3. APO-E.mp.
4. apolipoprotein e isoproteins.mp.
5. apoe isoproteins.mp.
6. apo e isoproteins.mp.
7. Apoproteins E.mp.
8. APOE-epsilon2.mp.
9. APOE epsilon2.mp.
10. ApoE2.mp.
11. APOE-epsilon 2.mp.
12. APOE epsilon 2.mp.
13. Apo E-2.mp.
14. Apo E 2.mp.
15. APO E2.mp.
16. Apolipoprotein-epsilon2.mp.
17. Apolipoprotein epsilon2.mp.
18. Apolipoprotein E-2.mp.
19. Apolipoprotein E 2.mp.
20. apolipoprotein E2.mp.
21. APOE-epsilon3.mp.
22. APOE epsilon3.mp.
23. ApoE3.mp.
24. APOE-epsilon 3.mp.
25. APOE epsilon 3.mp.
26. APO E-3.mp.
27. Apo E 3.mp.
28. APO E3.mp.
29. Apolipoprotein-epsilon3.mp.
30. Apolipoprotein epsilon3.mp.
31. Apolipoprotein E-3.mp.
32. Apolipoprotein E 3.mp.
33. Apolipoprotein E3.mp.
34. apoprotein E3.mp.
35. APOE-epsilon4.mp.
36. APOE epsilon4.mp.
37. APOE4.mp.
38. APOE-epsilon 4.mp.
39. APOE epsilon 4.mp.
40. Apo E-4.mp.
41. Apo E 4.mp.
42. apo e4.mp.
43. Apolipoprotein-epsilon4.mp.
44. Apolipoprotein epsilon4.mp.
45. Apolipoprotein E-4.mp.
46. Apolipoprotein E 4.mp.
47. Apolipoprotein E4.mp.
48. 1 or 2 or 3 or 4 or 5 or 6 or 7 or 8 or 9 or 10 or 11 or 12 or 13 or 14 or 15 or 16 or 17 or 18 or 19 or 20 or 21 or 22 or 23 or 24 or 25 or 26 or 27 or 28 or 29 or 30 or 31 or 32 or 33 or 34 or 35 or 36 or 37 or 38 or 39 or 40 or 41 or 42 or 43 or 44 or 45 or 46 or 47
49. Alzheimer disease.mp.
50. Alzheimer.mp.
51. Alzheimer's.mp.
52. Alzheimer Dementia.mp.
53. Dementia Alzheimer.mp.
54. Alzheimer-Type Dementia.mp.
55. Dementia Alzheimer-Type.mp.
56. Primary Senile Degenerative Dementia.mp.
57. Dementia Senile.mp.
58. senile dementia.mp.
59. Dementia Alzheimer Type.mp.
60. Alzheimer Type Dementia.mp.
61. Senile Dementia Alzheimer Type.mp.
62. Alzheimer Type Senile Dementia.mp.
63. Dementia Primary Senile Degenerative.mp.
64. Disease Alzheimer.mp.
65. Alzheimer Disease Late Onset.mp.
66. Focal Onset Alzheimer's Disease.mp.
67. Familial Alzheimer Disease.mp.
68. Sporadic Alzheimer disease.mp.
69. Alzheimer Disease Early Onset.mp.
70. Early Onset Alzheimer Disease.mp.
71. alzeimer disease.mp.
72. 49 or 50 or 51 or 52 or 53 or 54 or 55 or 56 or 57 or 58 or 59 or 60 or 61 or 62 or 63 or 64 or 65 or 66 or 67 or 68 or 69 or 70 or 71
73. neuron*.mp.
74. synap*.mp.
75. 73 or 74
76. degenerat*.mp.
77. dysfunction*.mp.
78. death.mp.
79. impair*.mp.
80. loss.mp.
81. dysregulat*.mp.
82. prun*.mp.
83. phagocyt*.mp.
84. 76 or 77 or 78 or 79 or 80 or 81 or 82 or 83
85. 75 and 84
86. neurodegenerat*.mp.
87. neurotoxic*.mp.
88. synaptotoxic*.mp.
89. myelin*.mp.
90. demyelin*.mp.
91. 86 or 87 or 88 or 89 or 90
92. 85 or 91
93. 48 and 72 and 92

**Prion-like spread**
1. apolipoprotein E/
2. APOE.mp.
3. APO-E.mp.
4. apolipoprotein e isoproteins.mp.
5. apoe isoproteins.mp.
6. apo e isoproteins.mp.
7. Apoproteins E.mp.
8. APOE-epsilon2.mp.
9. APOE epsilon2.mp.
10. ApoE2.mp.
11. APOE-epsilon 2.mp.
12. APOE epsilon 2.mp.
13. Apo E-2.mp.
14. Apo E 2.mp.
15. APO E2.mp.
16. Apolipoprotein-epsilon2.mp.
17. Apolipoprotein epsilon2.mp.
18. Apolipoprotein E-2.mp.
19. Apolipoprotein E 2.mp.
20. apolipoprotein E2.mp.
21. APOE-epsilon3.mp.
22. APOE epsilon3.mp.
23. ApoE3.mp.
24. APOE-epsilon 3.mp.
25. APOE epsilon 3.mp.
26. APO E-3.mp.
27. Apo E 3.mp.
28. APO E3.mp.
29. Apolipoprotein-epsilon3.mp.
30. Apolipoprotein epsilon3.mp.
31. Apolipoprotein E-3.mp.
32. Apolipoprotein E 3.mp.
33. Apolipoprotein E3.mp.
34. apoprotein E3.mp.
35. APOE-epsilon4.mp.
36. APOE epsilon4.mp.
37. APOE4.mp.
38. APOE-epsilon 4.mp.
39. APOE epsilon 4.mp.
40. Apo E-4.mp.
41. Apo E 4.mp.
42. apo e4.mp.
43. Apolipoprotein-epsilon4.mp.
44. Apolipoprotein epsilon4.mp.
45. Apolipoprotein E-4.mp.
46. Apolipoprotein E 4.mp.
47. Apolipoprotein E4.mp.
48. 1 or 2 or 3 or 4 or 5 or 6 or 7 or 8 or 9 or 10 or 11 or 12 or 13 or 14 or 15 or 16 or 17 or 18 or 19 or 20 or 21 or 22 or 23 or 24 or 25 or 26 or 27 or 28 or 29 or 30 or 31 or 32 or 33 or 34 or 35 or 36 or 37 or 38 or 39 or 40 or 41 or 42 or 43 or 44 or 45 or 46 or 47
49. Alzheimer disease.mp.
50. Alzheimer.mp.
51. Alzheimer's.mp.
52. Alzheimer Dementia.mp.
53. Dementia Alzheimer.mp.
54. Alzheimer-Type Dementia.mp.
55. Dementia Alzheimer-Type.mp.
56. Primary Senile Degenerative Dementia.mp.
57. Dementia Senile.mp.
58. senile dementia.mp.
59. Dementia Alzheimer Type.mp.
60. Alzheimer Type Dementia.mp.
61. Senile Dementia Alzheimer Type.mp.
62. Alzheimer Type Senile Dementia.mp.
63. Dementia Primary Senile Degenerative.mp.
64. Disease Alzheimer.mp.
65. Alzheimer Disease Late Onset.mp.
66. Focal Onset Alzheimer's Disease.mp.
67. Familial Alzheimer Disease.mp.
68. Sporadic Alzheimer disease.mp.
69. Alzheimer Disease Early Onset.mp.
70. Early Onset Alzheimer Disease.mp.
71. alzeimer disease.mp.
72. 49 or 50 or 51 or 52 or 53 or 54 or 55 or 56 or 57 or 58 or 59 or 60 or 61 or 62 or 63 or 64 or 65 or 66 or 67 or 68 or 69 or 70 or 71
73. amyloid beta protein/
74. amyloid protein/
75. amyloid plaque/
76. "amyloid beta protein[1-40]"/
77. "amyloid beta protein[1-42]"/
78. amyloid precursor protein/
79. amyloid/
80. tau protein/
81. tau.mp.
82. phf.mp.
83. tangles.mp.
84. neurofibrillary tangle/
85. paired helical filament/
86. straight filament.mp.
87. tauopathy/
88. neurofibrill*.mp.
89. 73 or 74 or 75 or 76 or 77 or 78 or 79 or 80 or 81 or 82 or 83 or 84 or 85 or 86 or 87 or 88
90. prion*.mp.
91. move*.mp.
92. moving.mp.
93. dynamic*.mp.
94. propagat*.mp.
95. attenuat*.mp.
96. spread*.mp.
97. secret*.mp.
98. transfer*.mp.
99. transmiss*.mp.
100. progress*.mp.
101. Trans-synaptic*.mp.
102. seed*.mp.
103. infect*.mp.
104. tunnel*.mp.
105. conver*.mp.
106. aggregat*.mp.
107. 90 or 91 or 92 or 93 or 94 or 95 or 96 or 97 or 98 or 99 or 100 or 101 or 102 or 103 or 104 or 105 or 106
108. 48 and 72 and 89 and 107

**Database: Web of science**

**Inflammation**

#14 #13 AND #3  
#13 #12 OR #11 OR #10 OR #8 OR #7 OR #5 OR #4
#12 TS= (“complement*” OR “complement component 4” OR “complement component, c4” OR “component 4, complement” OR “component, c4 complement” OR “c4, complement” OR “c4 complement component” OR “complement 4”OR “complement c4” OR “C1q” OR “c1q, complement” OR “complement 1q” OR “complement c1q” OR “complement component 1q” OR “component 1q, complement” OR “C3” OR “c3, complement” OR “complement 3” OR “complement c3” OR “complement component 3” “component 3, complement” OR “interleukin*” OR “IL1β” OR IL1-β OR “Interleukin 1 beta” OR “Interleukin- 1 beta” OR “il-1 beta” OR “interleukin 1beta OR “interleukin-1 beta” OR “interleukin-1beta” OR “tumor necrosis factor alpha” OR “tumor necrosis factor-alpha” OR “tnf-alpha”OR “tnfalpha” OR “cachectin” OR “cachectin tumor necrosis factor” OR “cachectin-tumor necrosis factor” OR OR “tumor necrosis factor ligand superfamily member 2” OR “tnf superfamily, member 2” OR TNFα” OR “cytokine*” or “gliosis” OR “pro-inflammat*” OR “anti-inflammat*” OR “inflammat* NEAR/2 mediat*” OR “neuroinflammat*” OR “phagocyt*” OR“immun*”)
#11 #9 AND #4
#10 #9 AND #5
#9 ts=("Reactiv*")
#8 #6 AND #4
#7 #6 AND #5
#6 TS= ("Activat*")
#5 TS= ("microglia" OR "cell microglia" OR "compound granular corpuscle" OR "glitter cell" OR "hortega cell" OR "microglia cell" OR "microglial cell" OR "neuromicroglia cell" OR "glia cell")
#4 TS= ("astro*" OR "astroglia cell" OR "astroglial cell" OR "glia cell" OR "type 1 astrocytes" OR "type 2 astrocytes")
#3 #2 AND #1
#2 TS= ( "Alzheimer" OR "Alzheimer's" OR “Alzheimer Dementia” OR "Dementia Alzheimer" OR "Alzheimer-Type Dementia" OR "Dementia Alzheimer-Type" OR "Primary Senile Degenerative Dementia" OR "Dementia Senile" OR “Senile Dementia” OR "Dementia Alzheimer Type" OR “Alzheimer Type Dementia” OR "Senile Dementia Alzheimer Type" OR “Alzheimer Type Senile Dementia” OR "Dementia Primary Senile Degenerative" OR “Alzheimer's Disease” OR "Disease Alzheimer" OR “Acute Confusional Senile Dementia” OR "Senile Dementia Acute Confusional" OR "Alzheimer Disease Late Onset" OR "Late onset Alzheimer's Disease" OR "Alzheimer's Disease Focal Onset" OR “Focal Onset Alzheimer's Disease” OR “Familial Alzheimer Disease” OR "Sporadic Alzheimer disease" OR "Alzheimer Disease Early Onset" OR “Early Onset Alzheimer Disease” OR "Alzheimer disease" OR "Alzeimer disease" OR "Alzeimer's disease" OR "Alzheimer dementia" OR "alzheimer sclerosis" OR "alzheimer syndrome" OR "alzheimer's disease" OR "dementia, alzheimer")
#1 TS=("apolipoprotein E" OR "APOE" OR "apo-e" OR "apolipoprotein e isoproteins" OR "apoe isoproteins" OR "APO E" OR "apo e isoproteins" OR "Apoproteins E" OR "Apoprotein E" OR "APOE-epsilon2" OR "APOE epsilon2" OR "ApoE2" OR "APOE-epsilon 2" OR "APOE epsilon 2" OR "Apo E-2" OR "Apo E 2" OR "apo e2" OR "Apolipoprotein-epsilon2" OR "Apolipoprotein epsilon2" OR "Apolipoprotein E-2" OR "Apolipoprotein E 2" OR "APOE-epsilon3" OR "apoe epsilon3" OR "ApoE3" OR "APOE-epsilon 3" OR "APOE epsilon 3" OR "APO E-3" OR "Apo E 3" OR "APO E3" OR "Apolipoprotein-epsilon3" OR "Apolipoprotein epsilon3" OR "Apolipoprotein E-3" OR "Apolipoprotein E 3" OR "APOE-epsilon4" OR "APOE epsilon4" OR "APOE4" OR "APOE-epsilon 4" OR "APOE epsilon 4" OR "Apo E-4" OR "Apo E 4" OR "apo e4" OR "Apolipoprotein-epsilon4" OR "Apolipoprotein epsilon4" OR "Apolipoprotein E-4" OR "Apolipoprotein E 4" OR "apolipoprotein ε" OR "APOε" OR "apo-ε" OR "apolipoprotein ε isoproteins" OR "apoε isoproteins" OR "APO ε" OR "apo ε isoproteins" OR "Apoproteins ε" OR "Apoprotein ε" OR "APOE-ε2" OR "APOE ε2" OR "Apoε2" OR "APOE-ε2" OR "APOE ε 2" OR "Apo ε-2" OR "Apo ε 2" OR "apo ε2" OR "Apolipoprotein-ε2" OR "Apolipoprotein ε2" OR "Apolipoprotein ε-2" OR "Apolipoprotein ε 2" OR "APOE-ε3" OR "apoe ε3" OR "Apoε3" OR "APOE-ε3" OR "APOE ε 3" OR "APO ε-3" OR "Apo ε 3" OR "APO ε3" OR "Apolipoprotein-ε3" OR "Apolipoprotein ε3" OR "Apolipoprotein ε-3" OR "Apolipoprotein ε 3" OR "APOE-ε4" OR "APOE ε4" OR "APOε4" OR "APOE-ε 4" OR "APOE ε 4" OR "Apo ε-4" OR "Apo ε 4" OR "apo ε4" OR "Apolipoprotein-ε4" OR "Apolipoprotein ε4" OR "Apolipoprotein ε-4" OR "Apolipoprotein ε 4")

**Neurodegeneration**

#9 #8 AND #3

#8 #7 OR #6
#7 TS=(”Neurodegeneration” OR “Neurotoxic*” OR “Synaptotoxic*” OR “Myelin*”, “demyelin*”)
#6 #5 AND #4
#5 ts=(“degenerat*” OR “Dysfunction*” OR “Death” OR “Impair*” OR “loss” OR “Dysregulat*” OR “prun*” OR “phagocyt*”)
#4 ts=(“Neuron*” OR “synap*” )
#3 #2 AND #1
#2 TS= ( "Alzheimer" OR "Alzheimer's" OR “Alzheimer Dementia” OR "Dementia Alzheimer" OR "Alzheimer-Type Dementia" OR "Dementia Alzheimer-Type" OR "Primary Senile Degenerative Dementia" OR "Dementia Senile" OR “Senile Dementia” OR "Dementia Alzheimer Type" OR “Alzheimer Type Dementia” OR "Senile Dementia Alzheimer Type" OR “Alzheimer Type Senile Dementia” OR "Dementia Primary Senile Degenerative" OR “Alzheimer's Disease” OR "Disease Alzheimer" OR “Acute Confusional Senile Dementia” OR "Senile Dementia Acute Confusional" OR "Alzheimer Disease Late Onset" OR "Late onset Alzheimer's Disease" OR "Alzheimer's Disease Focal Onset" OR “Focal Onset Alzheimer's Disease” OR “Familial Alzheimer Disease” OR "Sporadic Alzheimer disease" OR "Alzheimer Disease Early Onset" OR “Early Onset Alzheimer Disease” OR "Alzheimer disease" OR "Alzeimer disease" OR "Alzeimer's disease" OR "Alzheimer dementia" OR "alzheimer sclerosis" OR "alzheimer syndrome" OR "alzheimer's disease" OR "dementia, alzheimer")
#1 TS=("apolipoprotein E" OR "APOE" OR "apo-e" OR "apolipoprotein e isoproteins" OR "apoe isoproteins" OR "APO E" OR "apo e isoproteins" OR "Apoproteins E" OR "Apoprotein E" OR "APOE-epsilon2" OR "APOE epsilon2" OR "ApoE2" OR "APOE-epsilon 2" OR "APOE epsilon 2" OR "Apo E-2" OR "Apo E 2" OR "apo e2" OR "Apolipoprotein-epsilon2" OR "Apolipoprotein epsilon2" OR "Apolipoprotein E-2" OR "Apolipoprotein E 2" OR "APOE-epsilon3" OR "apoe epsilon3" OR "ApoE3" OR "APOE-epsilon 3" OR "APOE epsilon 3" OR "APO E-3" OR "Apo E 3" OR "APO E3" OR "Apolipoprotein-epsilon3" OR "Apolipoprotein epsilon3" OR "Apolipoprotein E-3" OR "Apolipoprotein E 3" OR "APOE-epsilon4" OR "APOE epsilon4" OR "APOE4" OR "APOE-epsilon 4" OR "APOE epsilon 4" OR "Apo E-4" OR "Apo E 4" OR "apo e4" OR "Apolipoprotein-epsilon4" OR "Apolipoprotein epsilon4" OR "Apolipoprotein E-4" OR "Apolipoprotein E 4" OR "apolipoprotein ε" OR "APOε" OR "apo-ε" OR "apolipoprotein ε isoproteins" OR "apoε isoproteins" OR "APO ε" OR "apo ε isoproteins" OR "Apoproteins ε" OR "Apoprotein ε" OR "APOE-ε2" OR "APOE ε2" OR "Apoε2" OR "APOE-ε2" OR "APOE ε 2" OR "Apo ε-2" OR "Apo ε 2" OR "apo ε2" OR "Apolipoprotein-ε2" OR "Apolipoprotein ε2" OR "Apolipoprotein ε-2" OR "Apolipoprotein ε 2" OR "APOE-ε3" OR "apoe ε3" OR "Apoε3" OR "APOE-ε3" OR "APOE ε 3" OR "APO ε-3" OR "Apo ε 3" OR "APO ε3" OR "Apolipoprotein-ε3" OR "Apolipoprotein ε3" OR "Apolipoprotein ε-3" OR "Apolipoprotein ε 3" OR "APOE-ε4" OR "APOE ε4" OR "APOε4" OR "APOE-ε 4" OR "APOE ε 4" OR "Apo ε-4" OR "Apo ε 4" OR "apo ε4" OR "Apolipoprotein-ε4" OR "Apolipoprotein ε4" OR "Apolipoprotein ε-4" OR "Apolipoprotein ε 4")

**Prion-like spread**

#8 #7 AND #6 AND #3
#7 TS= ("Prion*" OR "move*" OR "Moving" OR "dynamic*" OR "Propagat*" OR "Attenuat*" OR "Spread*" OR "Secret*" OR "Transfer*" OR "transmiss*" OR "progress*" OR "trans-synaptic*" or "seed*" or "conver*" or "infect*" or "tunnel*" or "aggregat*")
#6 #5 OR #4
#5 TS= ("tau" OR “τ” OR “PHF” OR "tau-protein" OR "microtubule associated protein tau" OR "microtubule protein tau" OR "tau proteins" OR "Neurofibrillary tangles" OR "tangles" OR "paired helical filament" OR "paired-helical filament" OR “Straight filament” OR "tauopathy" OR "neurofibrill*")
#4 TS= ("Amyloid" OR "Amyloido*" OR "Alzheimer's Amyloid Fibril Protein" OR "ABP Alzheimer's" OR "Alzheimer ABP" OR "Alzheimer's ABP" OR "Amyloid AD-AP" OR "AD-AP Amyloid" OR "Amyloid AD AP" OR "beta-Amyloid Protein" OR "Protein beta-Amyloid" OR "beta Amyloid Protein" OR "Amyloid beta-Protein" OR "Amyloid beta Protein" OR "beta-Protein Amyloid" OR "Amyloid Fibril Protein Alzheimer's" OR Amyloid Protein A4 OR "Protein A4 Amyloid" OR "Alzheimer beta-Protein" OR "Alzheimer beta Protein" OR "beta-Protein Alzheimer" OR "Amyloid beta-Peptide" OR "beta-Peptide Amyloid" OR "β-amyloid" OR "amyloid β" OR "Aβ" OR "Amyloid-β" OR "β-Amyloid Protein" OR "Protein β-Amyloid" OR "β Amyloid Protein" OR "Amyloid β-Protein" OR "Amyloid β Protein" OR "β-Protein Amyloid" OR "Alzheimer β-Protein" OR "Alzheimer βProtein" OR "β-Protein Alzheimer" OR "Amyloid -βPeptide" OR "β-Peptide Amyloid")
#3 #2 AND #1
#2 TS= ( "Alzheimer" OR "Alzheimer's" OR “Alzheimer Dementia” OR "Dementia Alzheimer" OR "Alzheimer-Type Dementia" OR "Dementia Alzheimer-Type" OR "Primary Senile Degenerative Dementia" OR "Dementia Senile" OR “Senile Dementia” OR "Dementia Alzheimer Type" OR “Alzheimer Type Dementia” OR "Senile Dementia Alzheimer Type" OR “Alzheimer Type Senile Dementia” OR "Dementia Primary Senile Degenerative" OR “Alzheimer's Disease” OR "Disease Alzheimer" OR “Acute Confusional Senile Dementia” OR "Senile Dementia Acute Confusional" OR "Alzheimer Disease Late Onset" OR "Late onset Alzheimer's Disease" OR "Alzheimer's Disease Focal Onset" OR “Focal Onset Alzheimer's Disease” OR “Familial Alzheimer Disease” OR "Sporadic Alzheimer disease" OR "Alzheimer Disease Early Onset" OR “Early Onset Alzheimer Disease” OR "Alzheimer disease" OR "Alzeimer disease" OR "Alzeimer's disease" OR "Alzheimer dementia" OR "alzheimer sclerosis" OR "alzheimer syndrome" OR "alzheimer's disease" OR "dementia, alzheimer")
#1 TS=("apolipoprotein E" OR "APOE" OR "apo-e" OR "apolipoprotein e isoproteins" OR "apoe isoproteins" OR "APO E" OR "apo e isoproteins" OR "Apoproteins E" OR "Apoprotein E" OR "APOE-epsilon2" OR "APOE epsilon2" OR "ApoE2" OR "APOE-epsilon 2" OR "APOE epsilon 2" OR "Apo E-2" OR "Apo E 2" OR "apo e2" OR "Apolipoprotein-epsilon2" OR "Apolipoprotein epsilon2" OR "Apolipoprotein E-2" OR "Apolipoprotein E 2" OR "APOE-epsilon3" OR "apoe epsilon3" OR "ApoE3" OR "APOE-epsilon 3" OR "APOE epsilon 3" OR "APO E-3" OR "Apo E 3" OR "APO E3" OR "Apolipoprotein-epsilon3" OR "Apolipoprotein epsilon3" OR "Apolipoprotein E-3" OR "Apolipoprotein E 3" OR "APOE-epsilon4" OR "APOE epsilon4" OR "APOE4" OR "APOE-epsilon 4" OR "APOE epsilon 4" OR "Apo E-4" OR "Apo E 4" OR "apo e4" OR "Apolipoprotein-epsilon4" OR "Apolipoprotein epsilon4" OR "Apolipoprotein E-4" OR "Apolipoprotein E 4" OR "apolipoprotein ε" OR "APOε" OR "apo-ε" OR "apolipoprotein ε isoproteins" OR "apoε isoproteins" OR "APO ε" OR "apo ε isoproteins" OR "Apoproteins ε" OR "Apoprotein ε" OR "APOE-ε2" OR "APOE ε2" OR "Apoε2" OR "APOE-ε2" OR "APOE ε 2" OR "Apo ε-2" OR "Apo ε 2" OR "apo ε2" OR "Apolipoprotein-ε2" OR "Apolipoprotein ε2" OR "Apolipoprotein ε-2" OR "Apolipoprotein ε 2" OR "APOE-ε3" OR "apoe ε3" OR "Apoε3" OR "APOE-ε3" OR "APOE ε 3" OR "APO ε-3" OR "Apo ε 3" OR "APO ε3" OR "Apolipoprotein-ε3" OR "Apolipoprotein ε3" OR "Apolipoprotein ε-3" OR "Apolipoprotein ε 3" OR "APOE-ε4" OR "APOE ε4" OR "APOε4" OR "APOE-ε 4" OR "APOE ε 4" OR "Apo ε-4" OR "Apo ε 4" OR "apo ε4" OR "Apolipoprotein-ε4" OR "Apolipoprotein ε4" OR "Apolipoprotein ε-4" OR "Apolipoprotein ε 4")
