# Supplementary material for: Invited Review: APOE at the interface of inflammation, neurodegeneration and pathological protein spread in Alzheimer's disease
Source: Neuropathol Appl Neurobiol. 2018 Nov 28;45(4):327–46. doi: 10.1111/nan.12529 (PMC6563457; doi:10.1111/nan.12529)
Supplement: Supplementary file 2 — Table S2. Exclusion Criteria [file NAN-45-327-s002.docx]

**Supplemental Information 2:** Exclusion Criteria

1. Not studying APOE in AD-related neurodegeneration, inflammation or pathological protein spread
2. Not studying AD (exclude other neurological diseases)
3. Not focused on the brain
4. Studies of APOE and Aβ or APOE and tau without studying protein spread
5. Human studies involving patients with mild cognitive impairment or animal studies using young APOE mice without additional AD pathology (e.g. no cross with tau or Aβ models or no lipopolysaccharide stimulation)
6. Human studies with evidence of mental health history (e.g. depression)
7. Evidence of neurological trauma (e.g. traumatic brain injury)
8. Evidence of infection (e.g. Herpes Simplex virus)
9. Studies of blood brain barrier dysfunction
10. Studies on olfactory system or retina
11. Studies of biomarkers
12. Reviews, editorials, case reports, case series studies, conference proceedings or posters
13. Duplicates
14. Studies not in English language
15. Studies where full text unavailable
