# Supplementary material for: Invited Review: APOE at the interface of inflammation, neurodegeneration and pathological protein spread in Alzheimer's disease
Source: Neuropathol Appl Neurobiol. 2018 Nov 28;45(4):327–46. doi: 10.1111/nan.12529 (PMC6563457; doi:10.1111/nan.12529)
Supplement: Supplementary file 3 — Table S3. Papers included in the systematic review (Discussed in the Results sections) (* denotes hand‐picked papers included in the systematic literature search) [file NAN-45-327-s003.docx]

**Supplemental Table 3: Papers included in the systematic review (Discussed in the Results sections) (* denotes hand-picked papers included in the systematic literature search)**

| **First author (Date)** | **Inflammation/ Neurodegeneration/ Neurodegeneration and Inflammation** | **Study design** | **Main Findings** | **Reference** |
| --- | --- | --- | --- | --- |
| Agosta (2009) | Neurodegeneration | Human - MRI | AD *APOE4* carriers had greater atrophy in bilateral parietal cortex, right precuneus, hippocampus and middle frontal gyrus vs. non-carriers and controls. | [3] |
| Anderson (1998) | Neurodegeneration | *In vivo* | APOE KO and WT mice had similar synaptic and dendritic densities. | [57] |
| Andrews-Zwilling (2010) | Neurodegeneration | *In vivo* | Female APOE4-KI mice had age-dependent decrease in hilar GABAergic interneurons vs. APOE3-KI. In neurotoxic APOE4 fragment transgenic mice, interneuron loss was even more pronounced. | [43] |
| Arendt (1997) | Neurodegeneration | Human - PM | AD *APOE4* carriers had more severe neurodegeneration in all areas investigated vs. non-carriers. Carriers had less plastic dendritic changes. Number of *APOE4* alleles influenced the pattern of dendritic arborisation. | [33] |
| Belinson (2008) | Neurodegeneration | *In vivo* | In APOE4-TR mice, activation of the amyloid cascade via inhibition of neprilysin resulted in degeneration of hippocampal CA1, entorhinal and septal neurons. This was accompanied by accumulation of intracellular Aβ and APOE along with lysosomal activation. | [39] |
| Belinson *(*2010) | Neurodegeneration | *In vivo* | In APOE4-TR mice, activation of the amyloid cascade via inhibition of neprilysin resulted in Aβ, oAβ and apoE4 co-localising with enlarged lysosomes and mitochondrial pathology. Kinetics of the lysosomal effects paralleled CA1 neuronal loss. | [72] |
| Belinson & Michaelson (2009) | Neurodegeneration & Inflammation | *In vivo* | APOE4-related CA1 neurodegeneration was associated with activation of astrogliosis and microgliosis. Microgliosis occurred earlier and was more prolonged compared to astrogliosis. Neurodegeneration within the septum was not associated with microgliosis or astrogliosis in either APOE3 or APOE4 mice. | [90] |
| Bien-Ly (2011) | Neurodegeneration | *In vivo* | C-terminal truncated APOE4/AD mice had greater Aβ levels and Aβ deposition, and displayed neuronal deficits such as reduced MAP2, calbindin and Fos immunoreactivity vs. AD mice expressing full length APOE3 or APOE4. | [78] |
| Blain (2006) | Inflammation | *In vivo* | Impairment in reactive sprouting in hE4 mice compared to hE3 mice. hE4 mice had more reactive astrocytes as well as a defective outward migration pattern of the astrocytes in the dentate gyrus. The expression of the anti-inflammatory cytokine IL-1ra was delayed in hE4 mice compared to hE3 mice. presence of apoE4 delays the astroglial repair process and indirectly compromises synaptic remodeling. | [114] |
| Blennow (1996) | Neurodegeneration | Human - PM | Rab3a levels were reduced in AD in hippocampus and frontal cortex, but not in cerebellum. No significant differences in rab3a levels in any brain region between AD patients possessing different numbers of the *APOE4* allele. | [52] |
| Boccardi (2004) | Neurodegeneration | Human - MRI | Greater atrophy in medial temporal lobe of AD *APOE4* carriers vs. non-carriers. | [11] |
| Brown (2002) | Inflammation | *In vitro* | Macrophages from male APOE4/4-TR mice produced significantly higher levels of nitric oxide than from male APOE3/3-TR mice, while macrophages from female APOE3/3-TR and female APOE4/4-TR mice produced the similar levels of nitric oxide. Primary cultures of microglial cells of APOE4 transgenic mice also produced significantly more nitric oxide than microglia from APOE3 transgenic mice. | [101] |
| Buttini (1999) | Neurodegeneration | *In vivo* | APOE3 protected against excitotoxin-induced neurodegeneration but APOE4 did not - seen as reduction in synaptophysin, MAP-2 positive neuronal dendrites and neurofilament positive axons. | [53] |
| Buttini (2000) | Neurodegeneration | *In vivo* | Hemizygous and homozygous APOE3 mice were protected against age-related and excitotoxin-induced neurodegeneration, but APOE4 mice were not. APOE3/E4 bigenic mice were as susceptible to neurodegeneration as APOE4 singly-transgenic mice. Neurodegeneration was more severe in homozygous than in hemizygous APOE4 mice consistent with a dose effect. | [38] |
| Buttini (2002) | Neurodegeneration | *In vivo* | In hAPP/APOE3 and hAPP/APOE mice, APOE3 but not APOE4, delayed age-dependent synaptic loss through a plaque-independent mechanism. | [54] |
| Buttini (2010) | Neurodegeneration | *In vivo* | Regardless of cellular source, APOE3 protected synapses and dendrites against excitotoxic injury, as did astrocytic APOE4. Neuronal APOE4 was associated with loss of neocortical and hippocampal pyramidal neurons, and increased fragmented APOE4. | [75] |
| Caberlotto (2016) | Inflammation | Computational | Identification of an alteration in aging-associated processes such as inflammation, oxidative stress and metabolic pathways. | [96] |
| Cambon (2000) | Neurodegeneration | *In vivo* | In aging, only human APOE4 mice displayed a decrease in synapse per neuron ratio, accompanied by an increase in synaptic size when compared with human APOE2, APOE KO and WT mice. | [49] |
| Camicioli (1999) | Neurodegeneration | Human - PM | In AD, neuron loss in the substantia nigra was associated with possession of *APOE4* allele, but not in nucleus basalis of Meynert or CA1 region of the hippocampus. | [34] |
| Chang (2005) | Neurodegeneration | *In vitro* | Fragments of APOE4 were neurotoxic, but full-length APOE4 was not. The lipid- and receptor-binding regions in APOE4 fragments act together to cause dysfunction and neurotoxicity. | [77] |
| Chen (2005) | Inflammation | *In vitro* | APOE4 but not APOE3 with presence of lipoprotein HDL or serum stimulated an inflammatory response in microglia by release of PGE2 & IL-1β. | [102] |
| Chung (2016) | Neurodegeneration & Inflammation | *In vitro* & *in vivo* | Synaptosome phagocytosis assays on APOE TR astrocytes. | [121] |
| Colton (2002) | Inflammation | *In vitro* | Significantly more NO was produced in *APO*E4 mice compared to *APO*E3 transgenic mice. | [100] |
| Corey-Bloom (2000) | Neurodegeneration | Human - PM | No correlation between *APOE4* allele dosage and synapse loss in AD. | [51] |
| Cudaback (2011) | Inflammation | *In vitro* | Activation of ATP or C5a complement receptor on microglia, causes microglial migration following APOE3>APOE2=APOE4. | [115] |
| Cudaback (2015) | Inflammation | *In vitro* & human | TLR4/LPS- and TLR3/Poly I:C-induced production of chemokine CCL3 followed APOE3< APOE2 = APOE4. The APOE expression levels followed apoE2>apoE3> apoE4. Post-mortem AD brains homozygous for APOE4 allele had increased CCL3 than those homozygous for APOE3. | [116] |
| Dorey (2017) | Inflammation | *In vitro* & *in vivo* | APOE4 promoted Aβ-induced neuroinflammation while APOE2 was protective. APOE4/AD mice had higher inflammatory cytokines than APOE2/AD mice. Lipidated APOE4 increased inflammation in astrocytes while recombinant APOE4 did not. Both forms of APOE2 offered protection against Aβ-induced neuroinflammation. | [105] |
| Drouet (2001) | Neurodegeneration | *In vitro* | APOE2 and APOE3 protected cortical neurons against apoptotic cell death induced by non-fibrillar Aβ. APOE4 had no protective effect. Effect involves interaction of APOE with C-terminal domain of Aβ1-40. | [42] |
| Drzezga (2009) * | Neurodegeneration | Human - MRI | No correlation between *APOE4* allele dose and grey matter loss in AD patients. | [25] |
| Dumanis (2009) | Neurodegeneration | *In vitro* & *in vivo* | APOE4-TR mice had reduced dendritic spine density and spine length compared to APOE2-TR and APOE3-TR mice in the cortex but not in hippocampus. | [64] |
| Egensperger (1998) | Inflammation | Human study | *APOE4* allele dependent increase in the number of activated microglia and the tissue taken up by activated microglia. | [86] |
| Filippini (2009) | Neurodegeneration | Human - MRI | In AD, grey matter volume decreased with increasing *APOE4* allele load in the bilateral medial and anterior temporal lobes. | [12] |
| Geroldi (1999) * | Neurodegeneration | Human - MRI | Smaller volumes with increasing dose of the *APOE4* allele in the hippocampus, entorhinal cortex and anterior temporal lobes in AD patients. Larger volumes in the frontal lobes with increasing *APOE4* alleles. | [17] |
| Gilmor (1999) | Neurodegeneration | Human - PM | Non-significant reduction in the number of cholinergic neurons in the nucleus basalis of Meynert in AD cases. No relationship *to APOE* allele status. | [35] |
| Gispert (2015) | Neurodegeneration | Human - MRI | *APOE4* carriers showed steeper hippocampal volume reductions with AD progression. *APOE4* carriers showed lower gray matter volume in the bilateral hippocampus, amygdala, parahippocampal cortex and temporal pole, the left angular and inferior parietal cortex, the right insula, the posterior cingulate and precuneus. | [13] |
| Gómez-Isla (1997) | Neurodegeneration | Human - PM | In AD, more than 50% of neurons were lost in the superior temporal sulus. Neither the amount nor the rate of neuronal loss correlated with *APOE* genotype. | [36] |
| Grouselle (1998) | Neurodegeneration | Human - PM | Somatostatin concentrations were significantly lower in patients carrying an *APOE4* allele. | [45] |
| Guo (2004) | Inflammation | *In vitro* | APOE3 and APOE4 blocked Aβ-induced proinflammatory response in activated glia. Without Aβ, APOE3 and APOE4 stimulate IL-1β levels in a concentration and isoform dependent manner, with APOE4 more effective than APOE3. | [103] |
| Harris (2003) | Neurodegeneration | *In vivo* & human | Ratios of APOE fragments to full-length APOE higher in AD cases vs. nondemented controls. AD and control cases with APOE4 had more APOE fragments than those without APOE4. APOE4 is more susceptible than APOE3 to proteolysis *in vitro*. Mice with high-level expression of C-terminal truncated APOE4 had neurodegeneration. | [76] |
| Hashimoto (2001) * | Neurodegeneration | Human - MRI | In AD patients, hippocampal volume decreased with increasing number of *APOE4* alleles. Amygdala volume also decreased with increasing *APOE4* gene dose. Whole brain volume increased with increasing *APOE4* gene dose. | [19] |
| Heinonen (1995) | Neurodegeneration | Human - PM | Synaptophysin-like immunoreactivity did not differ significantly in AD patients with or without and *APOE4* allele. | [50] |
| Holtzman (2000) | Neurodegeneration | *In vivo* | Plaque-associated neuritic dystrophy developed in App(V717F) TG mice expressing mouse or human APOE. Formation of dystrophic neurites required APOE. More fibrillar deposits and neuritic plaques were observed in APOE4-expressing App(V717F) mice vs. those with APOE3. | [62] |
| Hu (1998) | Inflammation | *In vitro* | In 3 experimental paradigms APOE inhibited Aβ-induced astrocytic activation. No APOE isoform-specific effects were found. | [104] |
| Hudry (2013) | Neurodegeneration | *In vivo* | In APP/PS1 mice, APOE4 increased oAβ, plaques, peri-plaque synapse loss, and dystrophic neurons compared to APOE3. Greater reduction in presynaptic synaptophysin near plaques with APOE3 and APOE4, but not APOE2. Post-synaptic proteins were unchanged with APOE2 and APOE3, but APOE4 had greater PSD95 loss near plaques. | [59] |
| Jack (1998) * | Neurodegeneration | Human - MRI | Hippocampal volumes were smaller in AD patients. Hippocampal volumes did not differ based on *APOE* genotype. | [24] |
| Jack (1998) * | Neurodegeneration | Human - MRI | Mean annual rate of hippocampal and temporal horn volume loss was greater in AD patients. *APOE* genotype was not associated with the annual rate of volume change. | [30] |
| Jain (2013) | Neurodegeneration | *In vivo* | Impairments in dendritic arborisation and a loss of spines in the hippocampus and entorhinal cortex of female NSE-APOE4 and APOE4-KI mice compared to their respective APOE3-expressing counterparts. NSE-APOE4 mice had more severe deficits in dendritic arborisation, spine density and morphology than apoE4-KI mice. GFAP-apoE4 mice did not have impairments in their dendrite arborisation or spine density and morphology. | [65] |
| Ji (2003) * | Neurodegeneration | *In vivo* & human | APOE4 mice had a lower density of dendritic spines than WT or APOE3 mice. In humans, APOE4 dose inversely correlated with dendritic spine density in AD. | [68] |
| Jórdan (1998) | Neurodegeneration | *In vitro* | APOE4 alone was toxic to cultures, whereas APOE3 had no effect. APOE3 treatment prevented the Aβ-induced toxicity. | [40] |
| Juottonen (1998) * | Neurodegeneration | Human - MRI | Greater volume loss in the entorhinal cortex of AD patients with an *APOE4* allele vs. those without an *APOE4* allele. This effect was especially prominent in females vs. males. | [18] |
| Kang (2018) * | Inflammation | *In vitro* & i*n vivo* | Transcriptomic study comparing sex, age, and isolation-method of microglia in multiple models of AD-like pathology (amyloid, tau, ageing, and inflammation). | [92] |
| Keren-Shaul (2017) * | Inflammation | *In vitro* & *in vivo* | Transcriptomic study (single-cell RNA seq) of microglia subsets in AD-like an mouse model. | [94] |
| Koffie (2012) | Neurodegeneration | *In vitro* & *in vivo* | Higher oAβ and synapse loss near plaques in AD *APOE4* carriers compared to *APOE3* carriers. Lipidated APOE4 co-localised with oAβ and increased synaptic localization of the oAβ. This required APOE receptors. | [60] |
| Krasemann (2017) | Neurodegeneration & Inflammation | *In vivo* & human | Identified a neurodegenerative phenotype of microglia after phagocytosis of apoptotic neurons which is driven by APOE-TREM2 pathway, this switches the microglia from homeostatic to disease-associated. | [93] |
| Laakso (2000) | Neurodegeneration | Human - MRI | Trend towards accelerated volume loss in the AD group vs. controls. No significant interactions between volume change and *APOE* status. | [31] |
| LaFerla (1997) | Neurodegeneration | Human - PM | Neuronal death, as shown by TUNEL, correlated with APOE uptake and intracellular Aβ stabilization. Cells with the most nuclear DNA fragmentation had the highest level of cell surface gp330 which binds APOE. | [71] |
| Lanz (2003) | Neurodegeneration | *In vivo* | Presence of human APOE2 in the APPSw+/- mice restored spine density to levels seen in Tg- controls. | [69] |
| Laskowitz (1998) | Inflammation | *In-vitro* | Brain cultures from APOE-deficient mouse pups showed enhanced NO production relative to cultures from wildtype mice and from transgenic mice expressing the human APOE3 isoform, demonstrating that endogenous APOE produced by glial cultures is capable of inhibiting microglial function. APOE produced within the brain may suppress microglial reactivity and thus alter the CNS response to acute and chronic injury. | [110] |
| Laskowitz (2001) | Inflammation | *In vitro* | APOE4 was less effective at reducing microglia activation and the release of TNFα and NO. Peptides from the APOE receptor binding region mimiced these effects, and deletions of the amino acids 146-149 abolished this effect. | [108] |
| Lehtovirta (1995) | Neurodegeneration | Human - MRI | AD patients with APO*E4/4* genotype had smaller volumes of the hippocampus and the amygdala than those with APO*E3/4* and those with *APOE3/3* or *APOE2/3.* Volumes of frontal lobes were similar across the AD subgroups. | [14] |
| Lehtovirta (1996) | Neurodegeneration | Human - MRI | AD *APOE4* carriers had the most extensive volume loss in the medial temporal lobe, hippocampus and amygdala compared to those without *APOE4.* This was greatest within *APOE4* homozygotes. | [15] |
| Leung (2012) | Neurodegeneration | *In vivo* | Age-dependent loss of hilar GABAergic interneurons, whereby GAD67- or somatostatin-positive–but not NPY- or parvalbumin-positive–interneuron loss was exacerbated by APOE4. This effect was sex-dependent. | [44] |
| Lin (2018) * | Neurodegeneration & Inflammation | *In vitro* - iPSCs & gene editing | Generated iPSCs of neurons, astrocytes and microglia from *APOE3* and *APOE4* individuals that were genetically engineered with Crispr-Cas9 to correct the *APOE* genotype and did transcriptomics on the altered cell types. | [95] |
| Liu (2010) * | Neurodegeneration | Human - MRI | AD *APOE4* carriers had smaller volume in hippocampus and amygdala. | [21] |
| Liu (2015) | Neurodegeneration | *In vivo* | Greater age-induced reduction in PSD95, drebrin and NMDAR subunits in the APOE4/FAD and 5xFAD/APOE-KO mice compared with APOE2/FAD and APOE3/FAD mice. | [61] |
| Liu (2017) | Inflammation | *In vivo* | APOE isoforms differentially affect amyloid plaque-associated neuroinflammation. APOE4 expression increased whereas APOE3 reduced amyloid-related gliosis in the mouse brains. | [89] |
| Lo (2011) * | Neurodegeneration | Human - MRI | *APOE4* in AD patients accelerated te the hippocampal atrophy. | [28] |
| Lupton (2016) | Neurodegeneration | Human - MRI | *APOE4* was associated with reduced hippocampal and amygdala volume in AD patients. | [16] |
| Lynch (2001) | Inflammation | *In vivo* & *in vitro* | APOE downregulates CNS production of TNFa, Il-1b, and Il-6 mRNA following stimulation with lipopolysaccharide (LPS). | [111] |
| Maezawa (2006a) | Inflammation | *In vitro* | Astrocyte stimulation and production of inflammatory cytokines. | [112] |
| Maezawa (2006b) | Neurodegeneration & inflammation | *In vitro* | Most paracrine-mediated neurodegeneration was due to microglia not astrocytes. Microglial damage to neurons followed TR-APOE4>TR-APOE3>TR-APOE2. Microglial p38MAPK-dependent cytokine secretion followed a similar pattern. | [106] |
| Manelli (2007) | Neurodegeneration | *In vitro* | Dose-dependent neurotoxicity was induced by oAβ with a ranking order of apoE4-TR > KO = apoE2-TR = apoE3-TR > WT. | [41] |
| Manning (2014) * | Neurodegeneration | Human - MRI | Hippocampal atrophy rates in *APOE4* carriers were significantly higher in AD compared with non-carriers. | [22] |
| Masliah (1995) | Neurodegeneration | *In vivo* | In APOE KO mice, there was an age-dependent loss of synaptophysin-immunoreactive nerve terminals and MAP2-immunoreactive dendrites in the neocortex and hippocampus, compared to controls. | [56] |
| McGeer (1997) | Inflammation | *In vitro* | APOE4 only significantly enhanced complement activation with Aβ. | [120] |
| Minett (2016) | Inflammation | Human - PM | *APOE2* allele was associated with expression of Iba1 and MSR-A, and *APOE4* with CD68, HLA-DR and CD64. | [85] |
| Mori (2002) * | Neurodegeneration | Human - MRI | *APOE4* dose was significantly correlated with the rate of hippocampal atrophy in AD. | [26] |
| Nathan (2002) | Neurodegeneration | *In vitro* | Cortical neurons from APOE KO mice have significantly shorter neurites than neurons from WT mice. Human APOE3 increased neurite outgrowth, whereas APOE4 decreased outgrowth dose-dependently. | [82] |
| Neustadtl (2017) | Neurodegeneration | *In vivo* | APOE4 mice have higher cortical calcineurin activity compared with APOE3 mice. Elevation in calcineurin associated with fewer dendritic spine number in layer II/III of the cortex. | [84] |
| Nwabuisi-Heath (2014) | Neurodegeneration | *In vivo* | During spine maintenance phase, density of GluN1 + GluA2 spines did not change with APOE2, while density of these spines decreased with APOE4 vs. APOE3, primarily due to the loss of GluA2 in spines. During spine loss phase, total spine density was lower in neurons with APOE4 compared to APOE3. | [63] |
| Ophir (2003) | Inflammation | *In vivo* | APOE3 (but not APOE4) transgenic mice have marked increased astrocyte activation 72 hrs after LPS. There was no effect on astrocytic proliferation only morphology. APOE4 had a similar phenotype to APOE-deficiency. | [91] |
| Ophir (2005) | Inflammation | *In vivo* | Expression of inflammation related genes were higher and more prolonged in apoE4 compared to apoE3 transgenic mice treated with LPS after 24hrs. Microglia activation and NF-βK regulated genes were higher in apoE4 than apoE3 transgenic mice. | [113] |
| Overmyer (1999) | Inflammation | Human - PM | *APOE4* carriers have more GFAP expression than those without *APOE4* allele. | [87] |
| Risacher (2010) | Neurodegeneration | Human - MRI | The presence of one or more *APOE4* alleles increased annual rate of atrophy in hippocampus and entorhinal cortex. | [29] |
| Rodriguez (2013) | Neurodegeneration | *In vivo* | Shorter dendrites and lower spine densities in basal shaft dendrites of APOE4 mice compared to APOE3 mice. Spine densities did not differ between APOE2 and APOE3. | [66] |
| Rodriguez (2014) | Neurodegeneration & Inflammation | *In vivo* | Cortical levels of IL-1β were higher in E4FAD mice compared to E3FAD mice. Increased microglial reactivity in E4FAD mice and higher density of reactive cells surrounding cortical plaques, than in E3FAD mice. No APOE dependent differences in microglia reactivity within the subiculum. | [88] |
| Scheff (2006) * | Neurodegeneration | Human - PM | AD patients had fewer synapses in the outer molecular layer of the dentate gyrus. This was not related to *APOE* genotype. | [46] |
| Scheff (2007) | Neurodegeneration | Human - PM | AD patients had fewer synapses in the stratum radiatum of the hippocampal CA1 subfield. This was not related to *APOE* genotype. | [47] |
| Scheff (2011) | Neurodegeneration | Human - PM | AD patients had fewer synapses in lamina 3 of the inferior temporal gyrus. This was not related to *APOE* genotype. | [48] |
| Schuff (2009) * | Neurodegeneration | Human - MRI | AD patients showed hippocampal volume loss over 6 months and accelerated loss over 1 year. Increased rates of hippocampal loss were associated with presence of the *APOE4* gene in AD. | [27] |
| Sen (2012) | Neurodegeneration | *In vitro* & *in vivo* | APOE3 but not APOE4, acts via LRP1 to protect synapses against oAβ by inducing PKCƐ synthesis. This was lipidation and APOE receptor dependent. | [70] |
| Shi (2017) | Neurodegeneration & inflammation | *In vitro* & *in vivo* | APOE4 causes more extensive atrophy and neurodegeneration but absence of APOE is protective. APOE4 also causes higher neuroinflammation while APOE-KO was protective. | [9] |
| Susanto (2015) | Neurodegeneration | Human - MRI | In AD Aβ+ subjects, *APOE4* carriers had more severe atrophy of the medial temporal lobe and thalamus compared to non-carriers. | [23] |
| Tai (2015) | Inflammation | *In vitro* and animal model | APOE4 affected Aβ‐induced inflammatory receptor signaling, with increased detrimental (toll‐like receptor 4‐p38α) and reduced beneficial (IL‐4R‐nuclear receptor) pathways. oAβ induced TNF-α secretion which followed APOE-KO> APOE4> APOE3>APOE2. This was inhibited by TLR4 antagonists. | [107] |
| Tannenberg (2006) | Neurodegeneration | Human - PM | Presence of *APOE4* resulted in significantly lower levels of pre-synaptic proteins in AD cases. | [58] |
| Thangavel (2017) | Inflammation | Human - PM | APOE4 and glia maturation factor were colocalised within amyloid plaques of AD brains as well as in the activated astrocytes surrounding the plaques | [97] |
| Tolar (1999) | Neurodegeneration | *In vitro* | Truncated APOE and APOE peptide elicit an increase in intracellular calcium levels, followed by death of hippocampal neurons in culture | [80] |
| Tulloch (2018) * | Inflammation | Human - Methylation | Lower *APOE* DNA methylation in non-neuronal cells, mainly glia, in AD cases. | [99] |
| Ulrich (2018) * | Inflammation | *In vitro* & *in vivo* | APOE and plaque interaction in the APP/PS1 mouse AD-like model. | [109] |
| Veinbergs (1999) | Neurodegeneration | *In vivo* | Mice expressing human APOE4 exhibited dendritic alterations compared to APOE3 mice. However, both APOE3 and APOE4 mice had preserved density of synaptophysin-immunoreactive pre-synaptic terminals. | [55] |
| Veinbergs (2002) | Neurodegeneration | *In vitro* | APOE4-mediated neurotoxicity was associated with calcium dysregulation via increased influx through calcium channels and reduced clearance. Mediated by APOE receptors. | [73] |
| Vitek (2009) * | Inflammation | *In vitro* & *in vivo* | Characterising inflammatory responses of innate immune response in APOE TR mice | [98] |
| Wang (2005) | Neurodegeneration | *In vivo* | APOE4 mice displayed significantly reduced excitatory synaptic transmission and dendritic arborisation. Despite these changes there were no signs of gliosis, amyloid deposition or neurofibrillary tangles in these mice. | [67] |
| Wolk and Dickerson (2010) * | Neurodegeneration | Human - MRI | AD *APOE4* carriers exhibited greater medial temporal lobe atrophy, whereas non-carriers had greater frontoparietal atrophy. | [20] |
| Yasuda (1998) | Neurodegeneration | Human - MRI | Positive correlation between whole brain volume and number of *APOE4* alleles in AD. | [32] |
| Yin *(*2014) | Neurodegeneration | *In vivo* - MRI | Increased hippocampal and cortical atrophy in aged APOE4 mice compared to APOE3 mice. Increased soluble and insoluble Aβ in aged APOE4 mice. | [37] |
| Zhong (2008) | Neurodegeneration | *In vivo* | Arg-61 APOE mice (domain interaction) had no gross structural abnormalities or significant loss of neurons. Age-dependent loss of synaptophysin in neocortex and hippocampus and lower levels of the postsynaptic neuroligin-1. Fewer bassoon-immunoreactive presynaptic boutons in hippocampus vs. WT mice. No significant difference observed in the neocortex. No differences in total dendritic area. | [81] |
| Zhu (2012) | Neurodegeneration & Inflammation | *In vivo* | After LPS APOE4 mice had increased glial activation with higher levels of microglia and astrocytes, and higher prolonged cytokine release (IL-1β, Il-6, TNF-α) compared to APOE2 and APOE3 mice. APOE-KO mice were similar to APOE4. APOE4 caused greater synaptic protein loss measured by three synaptic markers; PSD-95, drebin, synaptophysin. | [119] |
