# Supplementary material for: Invited Review: APOE at the interface of inflammation, neurodegeneration and pathological protein spread in Alzheimer's disease
Source: Neuropathol Appl Neurobiol. 2018 Nov 28;45(4):327–46. doi: 10.1111/nan.12529 (PMC6563457; doi:10.1111/nan.12529)
Supplement: Supplementary file 4 — Table S4. Details of human post mortem cases used for Figures 2‐5. MRC BBN: Medical Research Council Brain Bank Number, AD: Alzheimer's disease, PM: post mortem. [file NAN-45-327-s004.docx]

**Supplementary Table 4.** Details of human post-mortem cases used for Figures 2-5. MRC BBN: Medical Research Council Brain Bank Number, AD: Alzheimer’s disease, PM: Post-mortem.

| MRC BBN | Control vs  AD | Age | Sex | APOE genotype | PM delay (hours) | Brain weight (g) | Brain pH | Braak Stage |
| --- | --- | --- | --- | --- | --- | --- | --- | --- |
| BBN 28402 | Control | 78 | M | ε3/ε3 | 49 | 1503 | 6.33 | I |
| BBN 29082 | Control | 79 | F | ε3/ε4 | 80 | 1339 | 5.96 | III |
| BBN 28771 | AD | 85 | M | ε3/ε3 | 91 | 1183 | 5.95 | VI |
| BBN 19690 | AD | 57 | M | ε3/ε4 | 58 | 1200 | 5.90 | VI |
